# Trends and forecasts of vitamin A deficiency burden in China, 1990–2035

**DOI:** 10.3389/fnut.2025.1658507

**Published:** 2025-08-26

**Authors:** Zhongming Ye, Junping Liu, Lijiao You, Yaoguo Han, Xiaodan Zhang, Ming Lei

**Affiliations:** ^1^Department of Critical Care Medicine, Seventh People's Hospital, Shanghai University of Traditional Chinese Medicine, Shanghai, China; ^2^Department of Traditional Medicine, Seventh People's Hospital, Shanghai University of Traditional Chinese Medicine, Shanghai, China

**Keywords:** Global Burden of Disease, vitamin A deficiency, China, incidence, forecast

## Abstract

**Objective:**

The purpose of this study was to analyze changes in the disease burden caused by vitamin A deficiency (VAD) in China from 1990 to 2021, based on the Global Burden of Disease (GBD) Database, and to predict the disease burden trend in 2035 using the Bayesian age-period-cohort (BAPC) model.

**Methods:**

Based on modeled estimates from the GBD 2021 database, this study used Joinpoint regression to calculate annual percentage change (APC) and assess long-term trends (1990–2021) in VAD burden. Disaggregated analysis quantified the contributions of population growth, population aging, and epidemiological changes. The BAPC model was used to predict incidence, prevalence, and DALYs for 2035.

**Results:**

From 1990 to 2021, VAD incidence and prevalence significantly declined across all age groups in China, most notably in children under 5 years (−6.23% average annual change). Disability-adjusted life years (DALYs) improved substantially for children and adolescents. However, DALYs rebounded among those aged 50–74 years. Significant age and sex disparities exist. Children under 5 years bear the heaviest burden. Women have a higher incidence and prevalence than men, while DALY rates are lower in men across all age groups. While overall trends in incidence and prevalence declined, DALYs showed recent increases. Decomposition analysis revealed that favorable epidemiological shifts primarily drove the decline in incidence and prevalence. Conversely, population aging increased the number of cases and DALYs. Projections for 2035 indicate continued declines in incidence, prevalence, and DALYs across all age groups. However, the rate of decrease is projected to be slower in middle-aged and older populations compared to younger groups.

**Conclusion:**

This study reveals the complex dynamics and shifting burden of VAD in China. It affirms past prevention successes while highlighting new challenges, particularly the increasing disease burden driven by population aging. To achieve “Healthy China 2030” goals, future strategies require a more refined, lifecycle approach focusing on vulnerable groups (children, pregnant women, and the elderly), strengthening surveillance and evaluation systems, and promoting research into pathogenesis and technological innovation to meet evolving public health needs.

## Introduction

Vitamin A deficiency (VAD) is a major public health problem worldwide and poses a serious threat to human health (1). VAD not only causes stunted growth and vision loss in children but also increases the risk of infection, making it a key risk factor in the Global Burden of Disease (1, 2).

Over the past few decades, with the development of the global economy and the improvement of healthcare, the epidemic trend of vitamin A deficiency has been curbed to some extent in some parts of the world (3). However, the disease burden of vitamin A deficiency continues to show significant regional differences and imbalances globally (4). Vitamin A deficiency remains a serious public health challenge in low- and middle-income countries, particularly in sub-Saharan Africa and South Asia. Poverty, food shortages, limited access to health care, and epidemics of infectious diseases in these areas exacerbate the burden of vitamin A deficiency.

In China, with the rapid development of the economy and the continuous improvement of public health policies, the status of vitamin A deficiency has also been significantly improved. However, due to the vast geographical area, large population, and differences in development levels between regions, the distribution and disease burden of vitamin A deficiency in different regions and populations in China still have certain complexities and particularities. In recent years, although prevention and control measures for vitamin A deficiency have achieved some results, there is still a relative lack of systematic research on changes in its disease burden and future trends.

The purpose of this study was to analyze the changes in disease burden caused by vitamin A deficiency in China from 1990 to 2021, based on the Global Burden of Disease (GBD) database, and to predict the trend of disease burden in 2035 using the Bayesian age-period-cohort (BAPC) model. This study will help fill the gap in the current research on the burden of vitamin A deficiency in China, provide a basis for the development of more scientific and precise public health strategies to further reduce the adverse effects of vitamin A deficiency on population health, and inform targeted interventions to achieve the Healthy China 2030 goal.

## Methods

### Data sources

This study is based on the GBD 2021 database, which provides detailed epidemiological data on 371 diseases and injuries in 204 countries and territories between 1990 and 2021. The GBD 2021 database aggregates data from numerous sources, including but not limited to health surveys, medical records, and epidemiological studies conducted in each country. However, due to differences in data availability and quality in different regions, the exact estimated sample size or number of sources for each region may vary. We use standardized epidemiological analysis methods to ensure the global comparability of data. These data are freely available through the Global Health Data Exchange website (5), and detailed methods and models have been described in previous reports (6). Specifically, we extracted data on vitamin A deficiency, including incidence, prevalence, and disability-adjusted life years (DALYs). Data are accessed and downloaded through the Global Health Data Exchange (GHDx) platform.^1^

### Important definitions

In this study, three core indicators—incidence, prevalence, and DALYs—were used to assess the disease burden of vitamin A deficiency (1). The incidence rate reflects the number of new cases of vitamin A deficiency per 100,000 population per year; prevalence indicates the proportion of existing cases in a population at a given point in time; and DALYs comprehensively quantify the years of life lost due to premature death and the years lived with disability caused by vitamin A deficiency, using the GBD standard disability weights for calculation. All indicators reported 95% uncertainty intervals (UIs) (7). This interval is an important statistical tool calculated by GBD through multiple sampling and correlation matrices when processing international data, taking into account differences in methods and missing data. It reflects global differences in data collection and processing methods, as well as the reality that data quality is affected by a variety of factors and is essential for assessing the reliability of data and comparing the results of studies. Confidence intervals are statistically determined (8) and are used to estimate the possible range of population parameters, calculated from the sample data. In the present study, we obtained incidence and prevalence data from the GBD database and used the UIs to identify uncertainties in these data to more accurately reflect the reliability of the findings.

### Joinpoint regression analysis

In this study, the joinpoint regression model was used to calculate the annual average percent change (AAPC) (9) to evaluate the long-term trend of vitamin A deficiency disease burden in China from 1990 to 2021. The model identifies trend turning points through piecewise linear regression and calculates the average rate of change over time, which has the advantage of being able to objectively reflect the non-linear change characteristics of disease burden (10). Specifically, a piecewise regression model was established after natural logarithmic transformation of incidence, prevalence, and DALYs rate, and the optimal number of turning points was determined by the Monte Carlo permutation test (significance level *α* = 0.05), and the Bayesian information criterion was used for model selection. After identifying the annual percentage change in each period, the overall AAPC and its 95% confidence interval were calculated by weighting over the time span (11). All analyses were performed using Joinpoint Software 4.9.0.0, developed by the National Cancer Institute (12), and to ensure the robustness of the results, we performed 1,000 bootstrap sampling trials of the uncertainty intervals of the GBD data and confirmed the stability of the turning points by sensitivity analysis, excluding 5% of the data. This method has been widely used in Global Burden of Disease studies, and its effectiveness has been validated in trend analyses of similar nutritional diseases (13).

### Decomposition analysis

Disaggregation analysis is the analysis of data using a robust decomposition method (14) attributing differences in incidence, deaths, and DALYs between two time points to changes in three independent factors: (a) changes in the age structure of the aging population, i.e., a shift to a larger group of older people, commonly referred to as population ageing; (b) changes in population size; and (c) changes in age-specific rates of epidemiological changes, reflecting the combined effect of all factors except age structure and population size (6). The decomposition method breaks down the changes in disease-related deaths and DALYs into factors caused by population aging (A), population growth (P), and age-specific rate changes (M). The disaggregation results include the absolute and relative contribution of each factor to disease-related deaths or changes in DALYs (15). Absolute contribution represents the number of deaths or DALYs attributable to disease, while relative contribution (i.e., “attributable ratio”) is calculated by dividing the number of attributed deaths or DALYs by the total number of deaths or DALYs in 1990 and multiplying by 100%. If the number of attributed deaths exceeds the total number of deaths in 1990, the proportion may exceed 100%.

### Bayesian age-period-cohort (BAPC) analysis

In this study, a Bayesian age-period-cohort (BAPC) model was used to predict the disease burden trend of vitamin A deficiency in China in 2035 (16). The model uses a hierarchical Poisson regression framework to simultaneously estimate age, period, and birth cohort effects. In the construction of the model, we applied a second-order random walk prior to the age and cohort effects to ensure smoothness, and the period effect uses a first-order autoregressive prior to deal with the time dependence. Based on the historical data from 1990 to 2021, the posterior distribution of parameters was generated by the Markov chain Monte Carlo algorithm, and the predicted incidence rate in 2035 and its 95% confidence interval were finally output.

## Results

### Changes in the disease burden caused by vitamin A deficiency in China from 1990 to 2021

From 1990 to 2021, the incidence and prevalence of vitamin A deficiency in all age groups in China showed a significant downward trend (Tables 1, 2), with the most significant decrease in children under 5 years of age. The incidence in this population decreased from 21,209.36 cases per 100,000 people in 1990 (95% UI: 16,462.17–26,028.74) to 2,972.57 cases per 100,000 people in 2021 (95% UI: 2,111.46–4,340.00), with an EAPC of −6.23% (95% CI: −6.40 to −6.05) and a simultaneous decrease in prevalence (EAPC: −6.23%). A similar trend was observed in other age groups, such as the ≥95-year-old age group, with an incidence of EAPC of −5.39%. Despite this trend, the disease burden in the young childhood group was still significantly higher in 2021 than in the other populations (e.g., the incidence was 10.4 times higher in the <5-year-old group than in the 95 + year-old group).

**Table 1:** Changes in the incidence of vitamin A deficiency in China from 1990 to 2021.

Age	1990	2021	1990–2021
	Incidence rate (95% UI per)	Incidence rate (95% UI per)	EAPCs (95% CI)
<5	21209.36 (16462.17, 26028.74)	2972.57 (2111.46, 4340.00)	−6.23 (−6.40, −6.05)
5–9	14587.01 (9569.15, 21551.48)	2792.12 (1631.59, 4438.74)	−5.25 (−5.52, −4.98)
10–14	14470.17 (8949.55, 20655.15)	2690.82 (1502.44, 4345.58)	−5.43 (−5.67, −5.20)
15–19	11747.39 (7025.63, 18568.75)	2360.61 (1342.63, 3912.65)	−5.11 (−5.21, −5.02)
20–24	12138.40 (7029.13, 19750.42)	2453.71 (1387.07, 4124.72)	−5.17 (−5.33, −5.02)
25–29	11316.16 (6509.16, 18563.39)	2329.42 (1297.60, 3801.13)	−5.13 (−5.32, −4.95)
30–34	10034.43 (5712.12, 16776.51)	2195.72 (1240.52, 3714.82)	−4.92 (−5.14, −4.70)
35–39	8492.08 (4611.18, 13901.11)	1875.38 (1052.62, 3053.11)	−4.88 (−5.03, −4.73)
40–44	7308.14 (3900.70, 12559.83)	1556.17 (805.20, 2525.25)	−4.95 (−5.10, −4.81)
45–49	6006.42 (3215.44, 10495.13)	1260.86 (653.62, 2110.20)	−4.97 (−5.10, −4.84)
50–54	4702.23 (2499.66, 8027.04)	943.88 (510.48, 1556.06)	−5.05 (−5.17, −4.93)
55–59	3548.12 (2033.44, 5925.80)	681.72 (372.74, 1150.09)	−5.14 (−5.23, −5.05)
60–64	2649.08 (1421.51, 4293.20)	502.27 (276.61, 822.94)	−5.15 (−5.24, −5.06)
65–69	2099.51 (1126.34, 3544.96)	371.34 (200.09, 599.21)	−5.31 (−5.42, −5.19)
70–74	1876.63 (1099.80, 3038.34)	299.92 (163.50, 494.68)	−5.58 (−5.72, −5.45)
75–79	1568.81 (885.92, 2571.30)	230.52 (126.94, 375.90)	−5.75 (−5.94, −5.55)
80–84	1558.53 (890.79, 2545.07)	235.11 (130.84, 398.71)	−5.66 (−5.87, −5.45)
85–89	1613.53 (849.00, 2750.54)	242.02 (127.78, 421.05)	−5.69 (−5.91, −5.47)
90–94	1669.47 (915.90, 2914.61)	256.51 (122.58, 458.52)	−5.56 (−5.81, −5.31)
95+	1716.86 (800.41, 3085.59)	285.30 (137.59, 518.85)	−5.39 (−5.62, −5.16)

**Table 2:** Changes in the prevalence of vitamin A deficiency in China from 1990 to 2021.

Age	1990	2021	1990–2021
	Prevalence rate (95% UI per)	Prevalence rate (95% UI per)	EAPCs (95% CI)
<5	21204.70 (16458.98, 26024.57)	2963.63 (2103.17, 4332.08)	−6.23 (−6.40, −6.06)
5–9	14583.25 (9565.17, 21547.07)	2785.76 (1623.55, 4431.45)	−5.26 (−5.53, −4.99)
10–14	14467.17 (8945.36, 20651.75)	2685.59 (1496.54, 4338.65)	−5.44 (−5.68, −5.20)
15–19	11744.87 (7022.89, 18566.50)	2355.66 (1337.42, 3907.87)	−5.12 (−5.21, −5.02)
20–24	12136.23 (7026.10, 19748.00)	2449.48 (1382.59, 4120.67)	−5.18 (−5.33, −5.02)
25–29	11314.38 (6507.44, 18561.29)	2325.98 (1293.27, 3797.87)	−5.14 (−5.32, −4.95)
30–34	10033.01 (5710.89, 16775.34)	2192.93 (1237.05, 3711.43)	−4.92 (−5.14, −4.70)
35–39	8490.97 (4609.99, 13900.04)	1873.24 (1050.48, 3050.35)	−4.89 (−5.04, −4.74)
40–44	7307.35 (3900.08, 12558.87)	1554.68 (803.99, 2524.05)	−4.95 (−5.10, −4.81)
45–49	6005.94 (3214.98, 10494.60)	1259.97 (652.70, 2109.56)	−4.97 (−5.10, −4.84)
50–54	4701.97 (2499.38, 8026.75)	943.38 (510.11, 1555.67)	−5.05 (−5.17, −4.94)
55–59	3548.00 (2033.32, 5925.71)	681.47 (372.43, 1149.84)	−5.14 (−5.23, −5.05)
60–64	2649.03 (1421.44, 4293.16)	502.18 (276.43, 822.83)	−5.15 (−5.24, −5.06)
65–69	2099.49 (1126.32, 3544.94)	371.38 (200.28, 599.19)	−5.31 (−5.42, −5.19)
70–74	1876.63 (1099.81, 3038.33)	300.05 (163.90, 494.66)	−5.58 (−5.72, −5.45)
75–79	1568.81 (885.91, 2571.31)	230.71 (127.59, 375.98)	−5.75 (−5.94, −5.55)
80–84	1558.53 (890.79, 2545.06)	235.27 (131.21, 398.99)	−5.66 (−5.87, −5.45)
85–89	1613.53 (849.00, 2750.54)	242.11 (127.92, 421.06)	−5.69 (−5.90, −5.47)
90–94	1669.47 (915.90, 2914.62)	256.56 (122.69, 458.58)	−5.56 (−5.81, −5.31)
95+	1716.86 (800.41, 3085.60)	285.33 (137.65, 518.85)	−5.39 (−5.62, −5.16)

DALYs with vitamin A deficiency in China showed a clear age-specific trend (Table 3). Significant improvements were observed in the group of children and adolescents: DALYs for children under 5 years of age decreased significantly from 47.58 per 100,000 in 1990 to 10.43 per 100,000 in 2021 (EAPC: −5.34%), and the 5–19 age group showed a similar downward trend. However, in contrast, the burden of DALYs in the 50–74 age group rebounded, with the average annual growth rate in the 55–59 age group reaching 0.60% (95% CI: 0.38–0.83).

**Table 3:** Changes in the DALYs of vitamin A deficiency in China from 1990 to 2021.

Age	1990	2021	1990–2021
	DALYs rate (95% UI per)	DALYs rate (95% UI per)	EAPCs (95% CI)
<5	47.58 (30.25, 69.35)	10.43 (6.24, 15.95)	−5.34 (−5.84, −4.83)
5–9	27.81 (16.43, 44.66)	6.52 (3.65, 10.50)	−5.14 (−5.38, −4.89)
10–14	20.37 (11.32, 33.12)	4.51 (2.53, 7.08)	−5.31 (−5.59, −5.03)
15–19	2.26 (1.15, 3.80)	2.68 (1.40, 4.37)	0.30 (−0.12, 0.72)
20–24	2.08 (1.09, 3.47)	2.21 (1.13, 3.63)	−0.05 (−0.52, 0.42)
25–29	2.01 (1.05, 3.35)	2.01 (1.02, 3.23)	−0.30 (−0.74, 0.13)
30–34	1.95 (1.03, 3.24)	1.87 (1.04, 2.92)	−0.40 (−0.77, −0.02)
35–39	1.94 (1.05, 3.16)	1.73 (0.95, 2.78)	−0.45 (−0.79, −0.11)
40–44	1.87 (1.02, 3.13)	1.66 (0.89, 2.77)	−0.32 (−0.61, −0.03)
45–49	1.78 (0.99, 2.97)	1.64 (0.89, 2.78)	0.09 (−0.01, 0.20)
50–54	1.63 (0.92, 2.62)	1.63 (0.90, 2.67)	0.46 (0.31, 0.61)
55–59	1.52 (0.89, 2.46)	1.61 (0.92, 2.59)	0.60 (0.38, 0.83)
60–64	1.55 (0.88, 2.60)	1.61 (0.89, 2.62)	0.43 (0.24, 0.62)
65–69	1.68 (0.87, 2.86)	1.67 (0.82, 2.80)	0.27 (0.12, 0.43)
70–74	1.72 (0.94, 2.76)	1.71 (0.88, 2.82)	0.20 (0.04, 0.36)
75–79	1.65 (0.94, 2.61)	1.60 (0.91, 2.62)	0.02 (−0.13, 0.17)
80–84	1.52 (0.86, 2.45)	1.43 (0.79, 2.27)	−0.14 (−0.27, −0.01)
85–89	1.32 (0.74, 2.14)	1.25 (0.67, 2.03)	−0.26 (−0.37, −0.16)
90–94	1.12 (0.59, 1.90)	1.02 (0.52, 1.77)	−0.46 (−0.55, −0.37)
95+	1.03 (0.44, 1.89)	0.90 (0.38, 1.70)	−0.56 (−0.73, −0.39)

### Age and gender differences in vitamin A deficiency in China, 1990–2021

**Figure 1:**
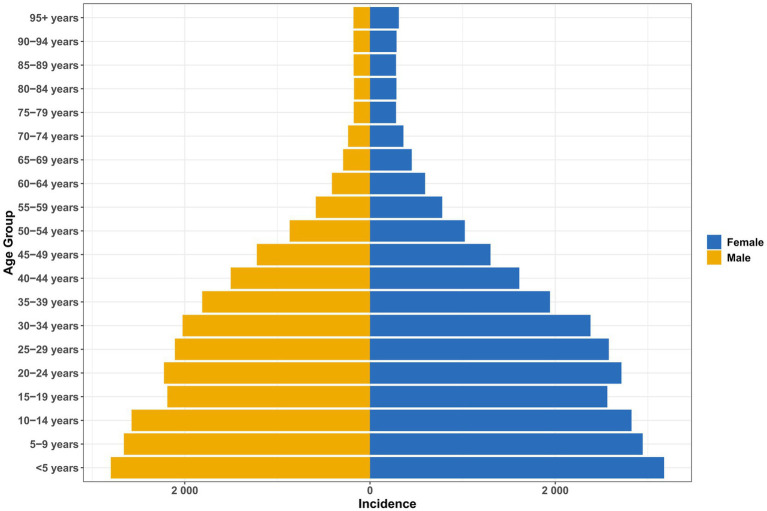
Age and sex differences in the incidence of vitamin A deficiency in China.

**Figure 2:**
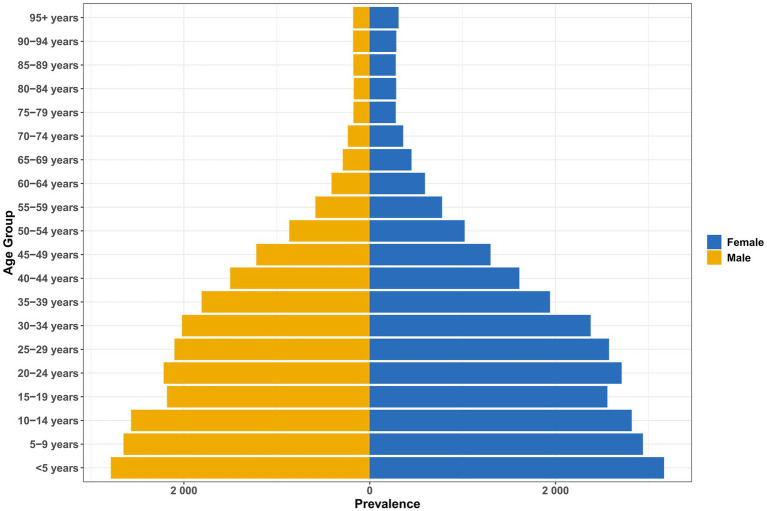
Age and sex differences in the prevalence of vitamin A deficiency in China.

From 1990 to 2021, there were significant age and sex differences in the incidence and prevalence of vitamin A deficiency in China (Figures 1, 2). Children under 5 years of age had the heaviest burden, and the incidence rate of female children (3174.76 cases/100,000; 95% UI: 1920.37–4999.91) and prevalence (3168.64/100,000) were significantly higher than those in male children (incidence: 2797.54/100,000; prevalence: 2786.16/100,000; Supplementary Table 1). This gender difference persisted into older age (e.g., prevalence was 310.50 per 100,000 women at 95 years versus 177.49 per 100,000 men; Supplementary Table 2), but it gradually decreased with age.

**Figure 3:**
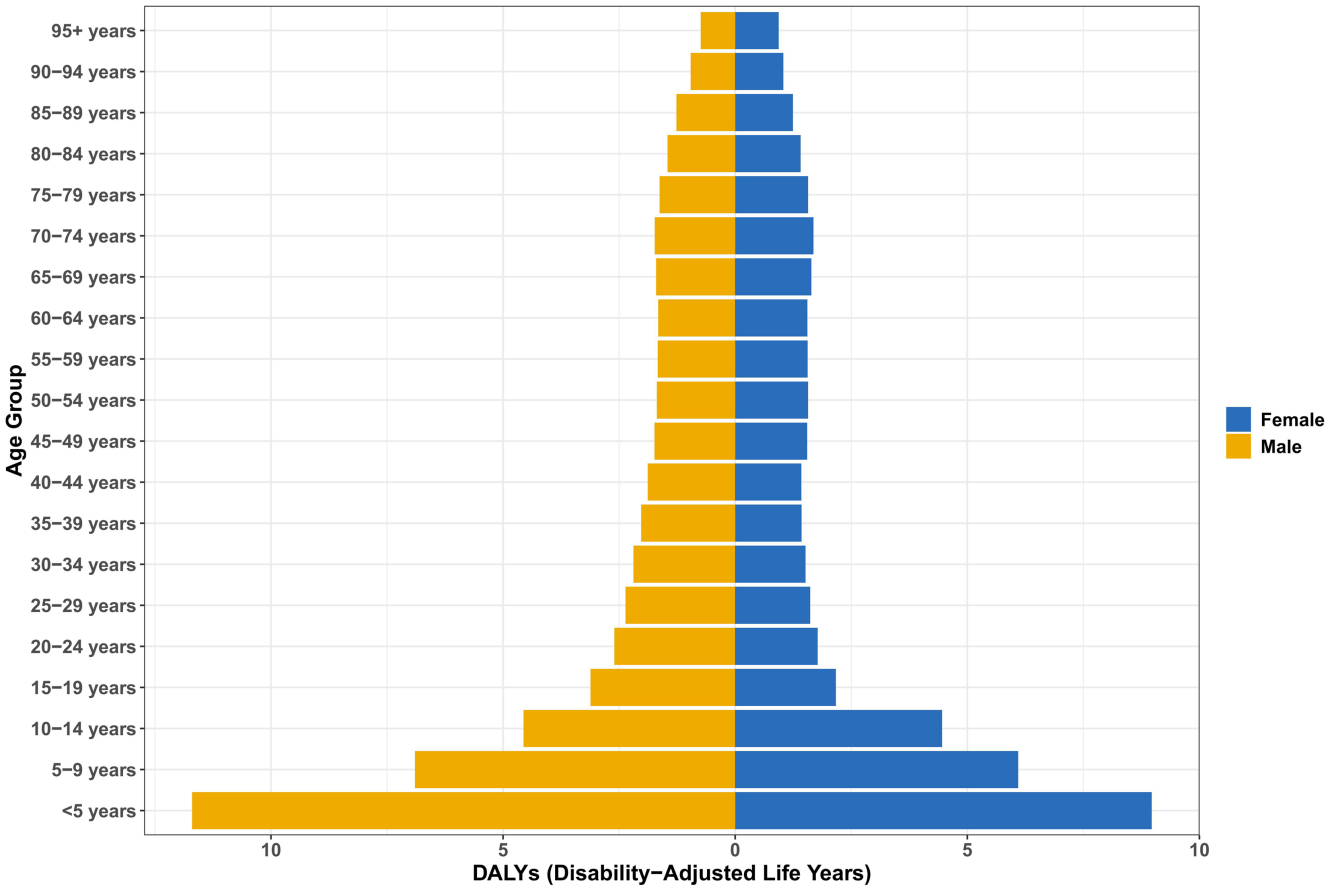
Age and sex differences in the DALYs of vitamin A deficiency in China.

Notably, the burden of DALYs showed gender reversal (Figure 3): although the incidence was higher in women, DALYs in men exceeded those in women across all age groups, especially in children under 5 years of age (11.70 per 100,000 men versus 8.97 per 100,000 women, a difference of 30.4%; Supplementary Table 3). The disease burden peaked in early childhood and then decreased sharply with age (e.g., 3.11 per 100,000 men aged 15–19 years), reaching its lowest level in old age (1.45 per 100,000 men aged 80–84 years).

### Analysis of annual percentage changes in vitamin A deficiency in China from 1990 to 2021

**Figure 4:**
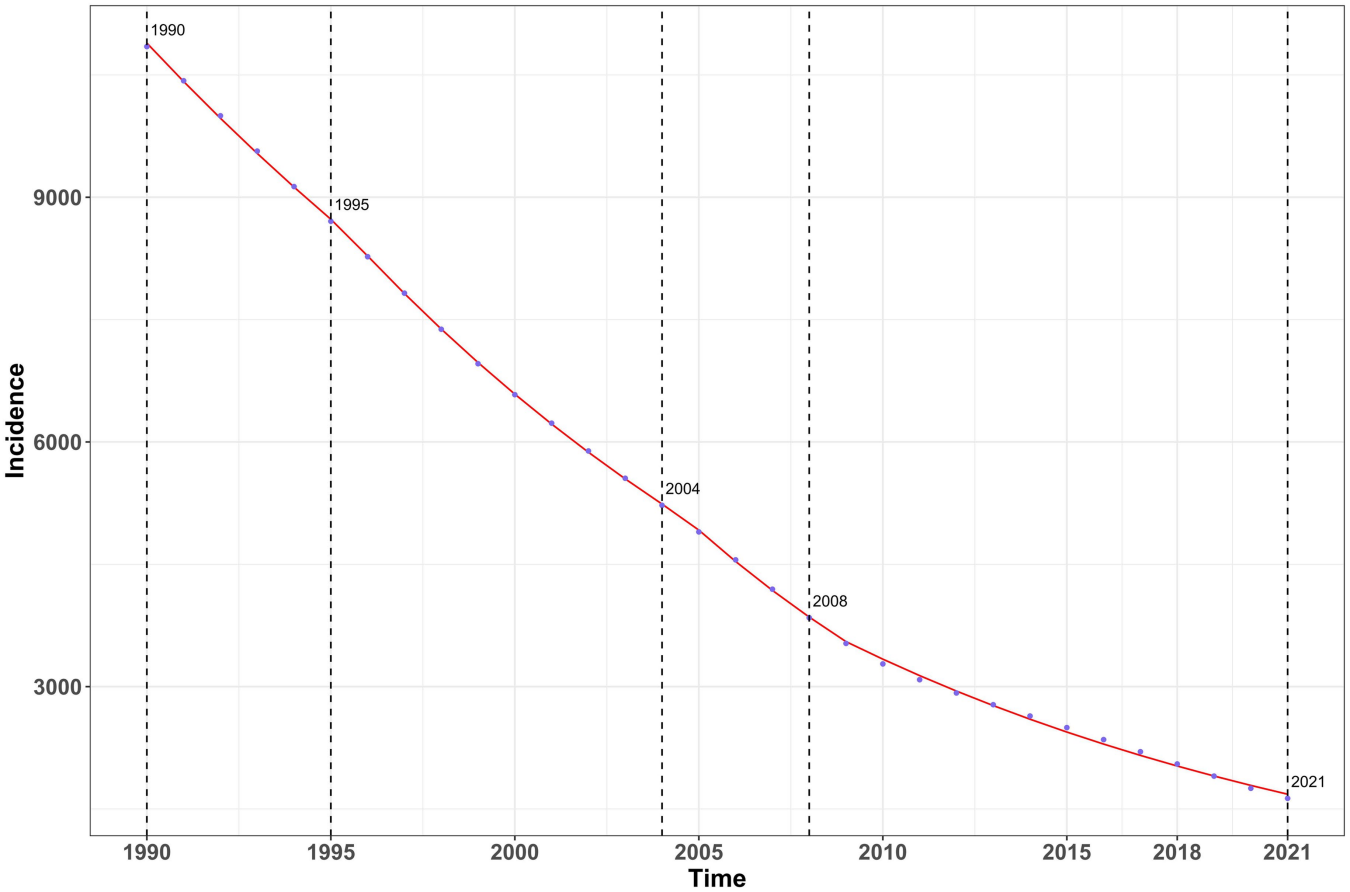
Analysis of annual percentage changes in incidence of vitamin A deficiency in China from 1990 to 2021.

**Figure 5:**
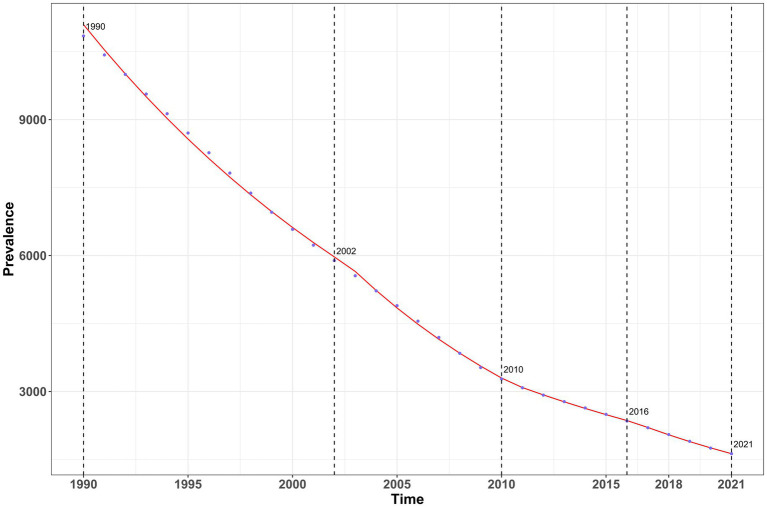
Analysis of annual percentage changes in Prevalence of vitamin A deficiency in China from 1990 to 2021.

The annual percentage change (AAPC) in the incidence, prevalence, and disability-adjusted life years (DALYs) of vitamin A deficiency in China from 1990 to 2021 showed an overall downward trend in the disease burden of vitamin A deficiency. In terms of incidence, there was a continuous downward trend during the study period (Figure 4), with the most significant decrease from 2004 to 2008 (AAPC = −7.82, 95%CI: −8.86 to −6.77; Supplementary Table 4); although the rate of decline slowed down after 2008 (AAPC = −6.04%), it remained at a high level. The trend of prevalence was basically the same as that of incidence but showed more obvious fluctuation characteristics (Figure 5) and decreased rapidly from 2002 to 2010 (AAPC = −7.41%; Supplementary Table 5), which showed a slight slowdown from 2010 to 2016 (AAPC = −5.23%) and then accelerated after 2016 (AAPC = −7.32%).

**Figure 6:**
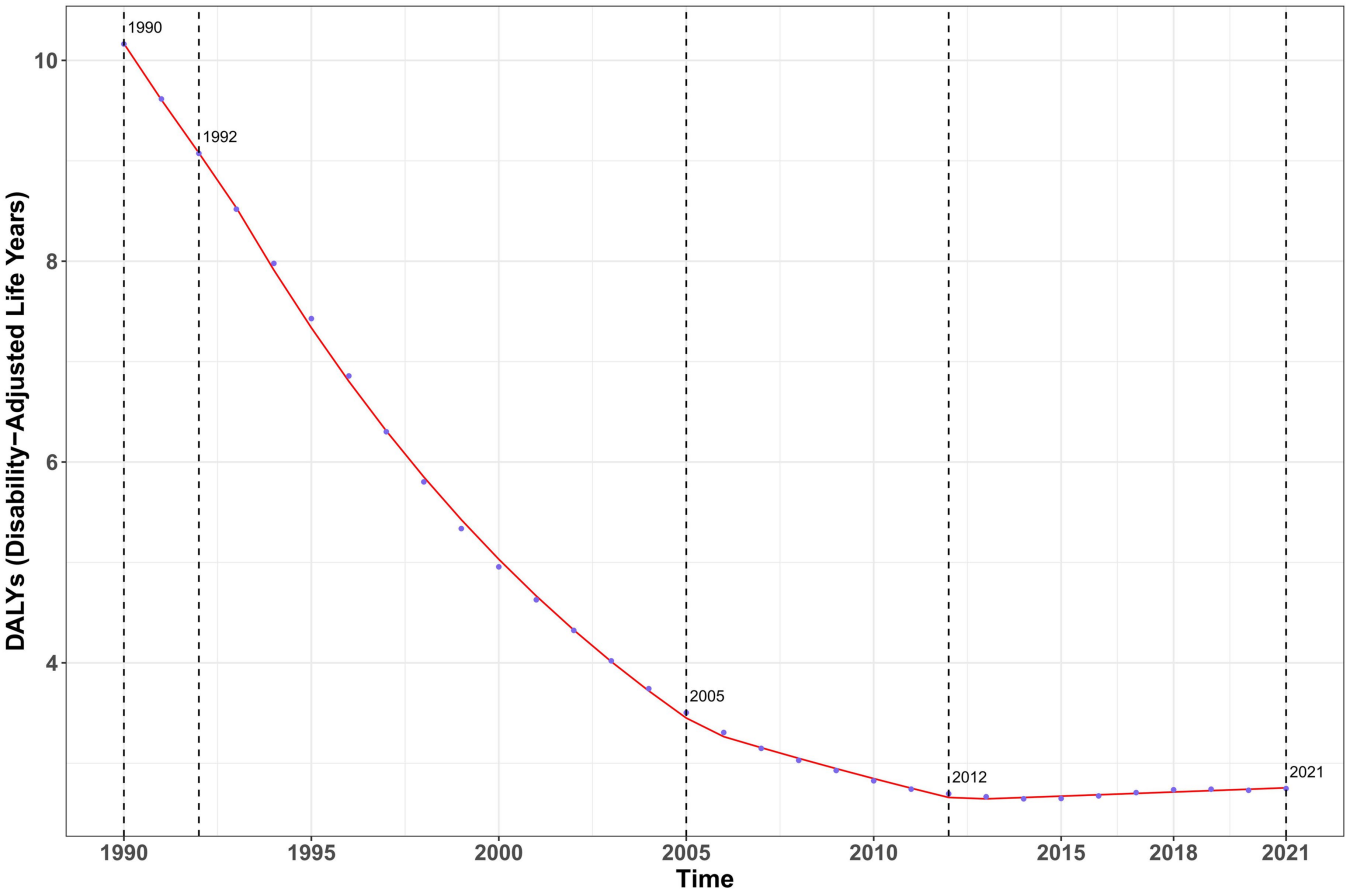
Analysis of annual percentage changes in DALYs of vitamin A deficiency in China from 1990 to 2021.

The analysis results of DALYs showed a more complex trend (Figure 6), with an overall decline from 1990 to 2012 and the most significant decline occurring from 1992 to 2005 (AAPC = −7.27%; Supplementary Table 6). However, after 2012, there was a trend reversal to an increase (AAPC = 0.51%). The annual percentage changes in all indicators were statistically significant (*p* < 0.001), and the incidence and prevalence continued to decrease, while DALYs showed an upward trend in recent years.

### A decomposition analysis of vitamin A deficiency in China from 1990 to 2021

**Figure 7:**
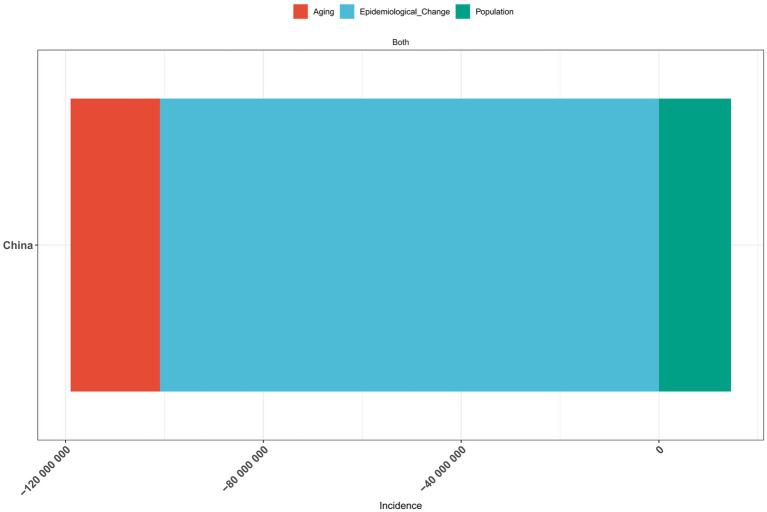
Decomposition analysis of vitamin A deficiency incidence in China, 1990–2021.

**Figure 8:**
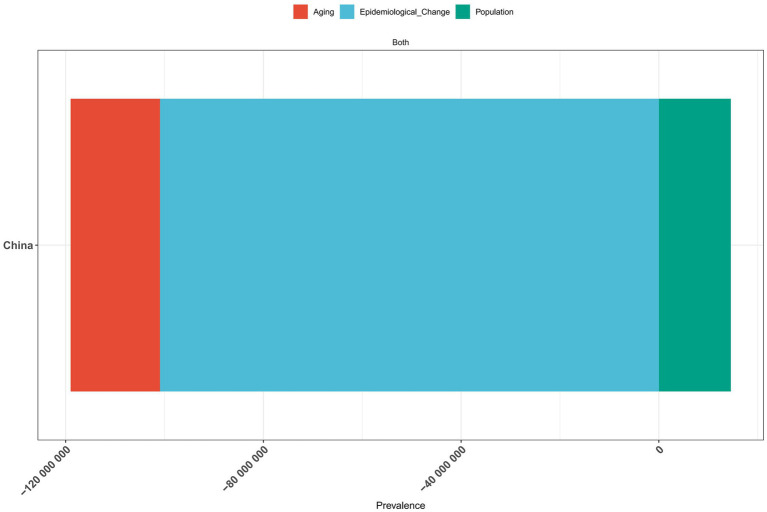
Decomposition analysis of vitamin A deficiency prevalence in China, 1990–2021.

**Figure 9:**
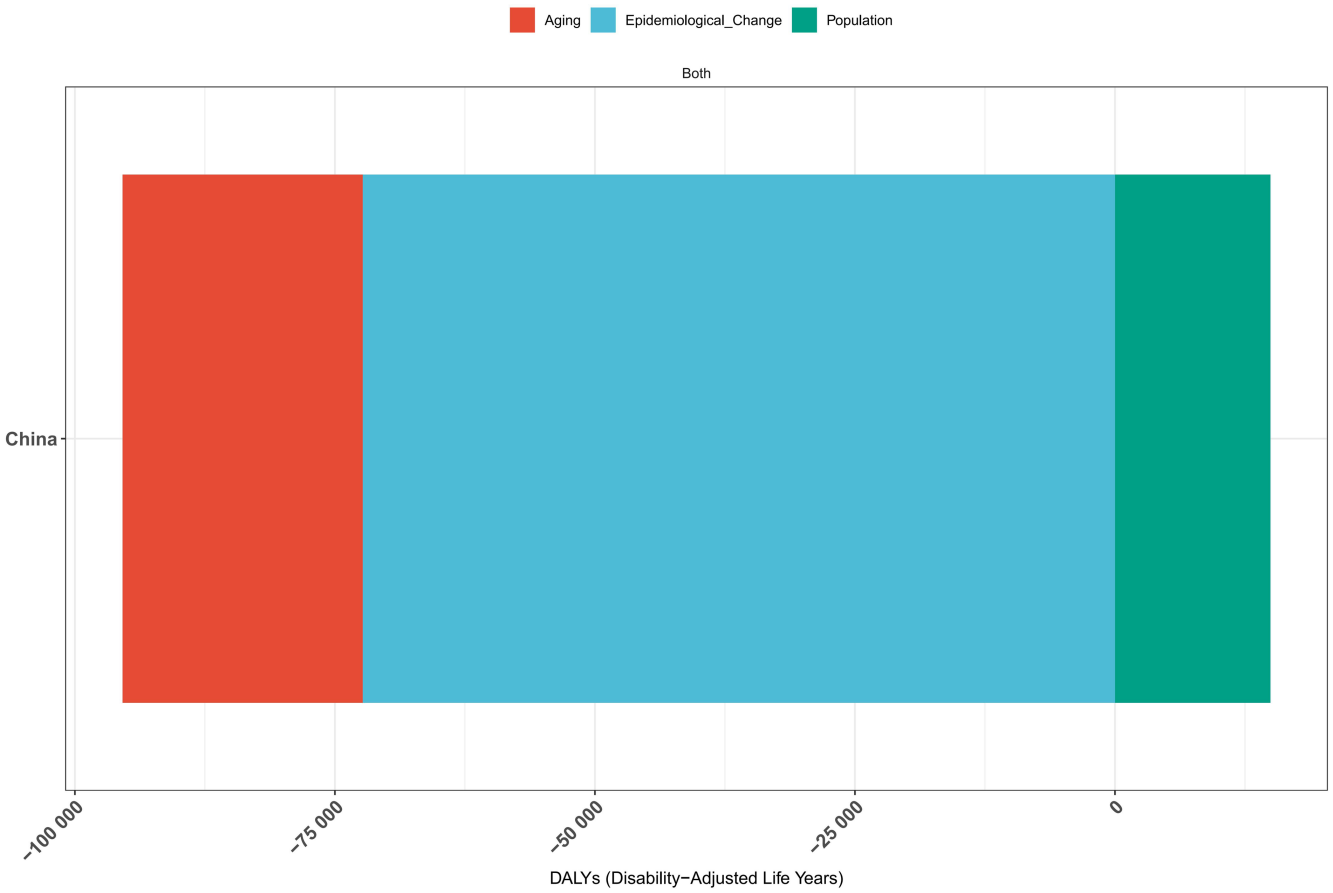
Decomposition analysis of vitamin A deficiency DALYs in China, 1990–2021.

The results of the decomposition analysis of vitamin A deficiency disease burden in China from 1990 to 2021 showed that there were significant differences in the effects of different influencing factors on disease indicators (Figures 7–9). In terms of incidence, epidemiological transition factors—reflecting the effectiveness of prevention and control measures—contributed the most significant decrease (−100.9 million cases, accounting for 96.63%; Supplementary Table 7), while population aging led to an increase of 18.07 million cases (−17.31%) and population growth contributed to an increase of 14.55 million cases (−13.94%). The combined effect of these three factors reduced the total incidence rate by 104.4 million cases. The pattern of change in prevalence was highly similar to that of the incidence rate. Among the total decrease of 104.4 million cases, epidemiological transition factors contributed 96.63% (−100.9 million cases; Supplementary Table 8). The proportions of aging (17.30%) and population growth (−13.93%) were consistent with the incidence rate. In terms of disability-adjusted life years (DALYs), epidemiological transition factors were still the main improvement factors (−72,300 DALYs, accounting for 89.88%; Supplementary Table 9), while the burden caused by aging increased more significantly (23,100 DALYs, accounting for 28.69%). Overall, total DALYs decreased by 80,500.

### Projection of the disease burden of vitamin A deficiency in China, 1990–2035

**Figure 10:**
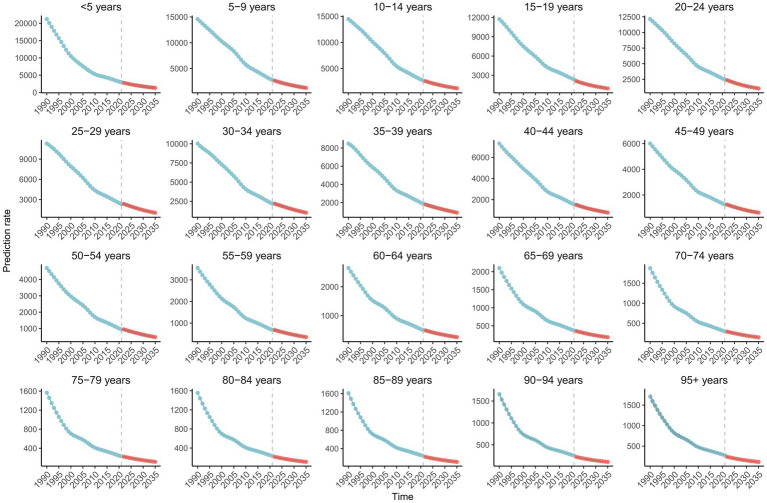
Projection of vitamin A deficiency incidence rate in China, 1990–2021 and forecast to 2035.

The disease burden projection of vitamin A deficiency in China from 1990 to 2035 shows that the incidence of vitamin A deficiency in China has shown a continuous downward trend in all age groups, but there are significant differences in the decline rate among different age groups (Figure 10; Supplementary Table 10). The incidence rate in the group of children under 5 years of age decreased significantly from 21,209.29 per 100,000 in 1990 to 2,972.56 per 100,000 in 2021 and is projected to further decrease to 1,330.69 per 100,000 in 2035, with an average annual decline rate (EAPC) of −5.34% (1990–2021). The downward trend in the adolescent group (5–19 years) was similar to that in the toddler group, where the incidence rate in the 10–14-year age group decreased from 14,470.23 per 100,000 people in 1990 to 2,690.83 per 100,000 people in 2021 and is expected to fall to 1,207.71 per 100,000 people in 2035. It is worth noting that, although the absolute incidence rate of the middle-aged and elderly group (≥50 years old) is low, the rate of decline is relatively slow, for example, the 55–59-year-old group has decreased from 3,548.14 per 100,000 people in 1990 to 681.74 per 100,000 people in 2021 and is predicted to be 351.80 per 100,000 people in 2035. The incidence rate in the elderly group (≥80 years) decreased most modestly, from 1,707.48 per 100,000 people in 1990 to 285.33 per 100,000 people in 2021 and is expected to be 114.17 per 100,000 people in 2035.

## Discussion

Based on the Global Burden of Disease database, this study comprehensively analyzed the epidemiological characteristics and changes in disease burden of vitamin A deficiency in China from 1990 to 2021, and predicted the disease trend in 2035. The results revealed the complex distribution pattern and dynamic trend of vitamin A deficiency in the Chinese population, which provided an important basis for public health intervention.

This study found that the incidence and prevalence of VAD in China showed a significant decreasing trend from 1990 to 2021, especially in children under 5 years of age, with an average annual decline rate of 6.23%. This positive change reflects not only the effectiveness of public health interventions in China but also the synergistic impact of key socioeconomic drivers, including national income growth (17), education popularization (18), and targeted poverty alleviation projects (19). It is worth noting that the downward trend showed obvious phased characteristics in different periods: the most significant decline was from 2004 to 2008 (AAPC = −7.82%), which coincided with the full implementation of the policy of equalization of basic public health services during the 11th Five-Year Plan (20). Access to child health services and the expansion of vitamin A supplementation programs may be key factors contributing to this rapid decline (21). However, we have also observed significant differences in the degree of improvement across age groups. Although the absolute incidence in children has decreased significantly, in 2021, the incidence in children under 5 years of age was still 10.4 times higher than in people over 95 years of age. This difference may be due to two factors: on the one hand, children have a relatively higher need for vitamin A and are more susceptible to infectious diseases that accelerate vitamin A consumption (22). On the other hand, the metabolism and utilization efficiency of vitamin A in the elderly population may have been adaptively altered (23). These findings suggest that we need to develop differentiated intervention strategies for different age groups.

The pattern of gender disparities in VAD revealed in this study is profound. The study found that women consistently had a higher incidence and prevalence than men but that men had a higher burden of DALYs, especially in the group of children under 5 years of age (30.4% higher in men than women). This seemingly paradoxical phenomenon can be explained from several perspectives: biologically, changes in estrogen levels in women may have an impact on vitamin A metabolism. Studies have shown (24) that estrogen may affect the balance of vitamin A in the body by regulating the absorption, storage, and utilization of vitamin A. For example, estradiol is predominantly metabolized by 2-hydroxylation in female rats (25), while in male rats, it is predominantly metabolized by 2- and 16α-hydroxylation (26). In addition, women’s vitamin A needs increase significantly during puberty, pregnancy, and lactation (27, 28). Studies have shown (29) that mothers need to deliver large amounts of vitamin A to the fetus during pregnancy, which can lead to a constant depletion of the mother’s vitamin A stores. The higher burden of DALYs in men (especially young boys) may stem from their inherent susceptibility to severe infections and immune response dysregulation due to vitamin A deficiency (30). Studies have shown (30, 31) that vitamin A deficiency in men may lead to excessive and poorly controlled inflammation, along with impaired tissue repair mechanisms, leading to more serious complications and exacerbating the health effects of vitamin A deficiency.

The results showed that the DALYs associated with vitamin A deficiency in China showed an upward trend after 2012 (AAPC = 0.51%), especially among the 50–74-year-old population. An in-depth analysis shows that this trend reversal is the result of multiple interacting factors, primarily including the following: (1) the significant impact of population aging: decomposition analysis quantitatively shows that aging contributed to 28.69% of the increase in DALYs. These data are in good agreement with physiological studies: vitamin A absorption is less efficient in older adults than in younger adults (32), and chronic inflammatory states accelerate vitamin A metabolic depletion (33). (2) Compounding effects of lifestyle changes: Rapid urbanization brings dual nutritional challenges. On the one hand, the increased consumption of refined foods has led to reduced dietary fiber intake, which may affect the bioavailability of *β*-carotene. On the other hand, the reduction in outdoor activity time leads to insufficient UV exposure, which affects the activation and metabolism of vitamin A (34). Studies have shown (35) that the average daily time spent outdoors by urban residents has decreased significantly. (3) Limitations of prevention and control strategies are as follows: Although epidemiological transformation factors—reflecting the effectiveness of prevention and control measures—contributed to 96.63% of the reduction in incidence, the current prevention and control system has obvious shortcomings: (1) uneven coverage of the target population: Existing interventions are mainly focused on children, and the vitamin A screening rate of adults is insufficient; (2) lag in response to dietary transformation: The proportion of animal retinol in a source of vitamin A in urban populations increased, while the proportion of plant-basedβ-carotene has correspondingly decreased; (3) lack of intervention for the elderly: Predictions showed that the rate of improvement in the ≥80-year-old group (EAPC = −2.1%) is significantly slower than that in children (EAPC = −5.3%). These findings suggest that the prevention and control of vitamin A deficiency in China should adopt a “whole life cycle” management strategy, focusing on (1) early screening of middle-aged and elderly populations and (2) precise dietary guidance (vitamin A supplementation regimen for urban and rural areas and age).

Projections for 2035 provide an important basis for policymaking. First, the incidence rate in children under 5 years of age is expected to continue to decline, but the absolute burden is still high (1330.69 per 100,000), suggesting that the current intervention needs to be maintained. Second, the improvement trend in the adolescent group (5–19 years old) indicates that the school’s feeding program has been effective, but there is still room for improvement. Third, the slow improvement trend in the middle-aged and older population, especially in the 55–59 age group (forecast 351.80 per 100,000 in 2035), calls for the development of targeted intervention strategies. Based on these findings, we suggest the following recommendations: (1) incorporate vitamin A deficiency prevention and control into the aging health strategy, (2) develop intervention programs for different age groups, and (3) strengthen the nutrition monitoring system throughout the life cycle.

There are the following limitations to this study: first, data from rural and remote areas may be insufficient, and the national aggregate data based on GBD do not cover provincial and municipal subdivision data or related individual’s socioeconomic status indicators. Therefore, the impact of socioeconomic gradients or regional differences on the burden of vitamin A deficiency cannot be quantitatively assessed. Second, the impact of dietary pattern changes was not fully considered. Finally, the adaptability of predictive models to public health emergencies is limited. Future research should (1) quantitatively assess the impact of regional differences and socioeconomic gradients on the burden of vitamin A deficiency using regionally stratified populations integrated with socioeconomic status variables, (2) explore the application of novel biomarkers, and (3) develop dynamic forecasting models.

## Conclusion

This study systematically depicts the 30-year (1990–2021) changes in vitamin A deficiency disease burden in China, revealing differences across three dimensions: age, gender, and period. The findings not only affirm the historic achievements in vitamin A deficiency prevention and control in China but also identify the new challenges facing the country, especially the shift in the disease burden caused by population aging. To achieve the strategic goal of “Healthy China 2030,” it is necessary to build a more refined, life-cycle vitamin A deficiency prevention and control system in the future. First, we should focus on vulnerable groups such as children, pregnant women, and the elderly. Second, it is necessary to strengthen the construction of disease surveillance and effect evaluation system. At the same time, it is necessary to carry out in-depth research on relevant pathogenesis and technological innovation to respond to the changing public health needs.

## Data Availability

The original contributions presented in the study are included in the article/Supplementary material, further inquiries can be directed to the corresponding authors.

## Footnotes

^1^ http://ghdx.healthdata.org/gbd-results-tool
