# Supplementary material for: Trends and forecasts of vitamin A deficiency burden in China, 1990–2035
Source: Front Nutr. 2025 Aug 26;12:1658507. doi: 10.3389/fnut.2025.1658507 (PMC12417177; doi:10.3389/fnut.2025.1658507)
Supplement: Supplementary file 1 [file Table_1.docx]

| Age and gender differences in the incidence of vitamin A deficiency in China from 1990 to 2021 | | | | | | | |
| --- | --- | --- | --- | --- | --- | --- | --- |
| Measure | Location | Sex | Age | Cause | Metric | Year | Value |
| Incidence | China | Male | <5 years | Vitamin A deficiency | Rate | 2021 | 2797.542235 |
| Incidence | China | Female | <5 years | Vitamin A deficiency | Rate | 2021 | 3174.759102 |
| Incidence | China | Male | 10-14 years | Vitamin A deficiency | Rate | 2021 | 2574.787689 |
| Incidence | China | Female | 10-14 years | Vitamin A deficiency | Rate | 2021 | 2823.579925 |
| Incidence | China | Male | 15-19 years | Vitamin A deficiency | Rate | 2021 | 2187.209673 |
| Incidence | China | Female | 15-19 years | Vitamin A deficiency | Rate | 2021 | 2561.547754 |
| Incidence | China | Male | 20-24 years | Vitamin A deficiency | Rate | 2021 | 2223.429451 |
| Incidence | China | Female | 20-24 years | Vitamin A deficiency | Rate | 2021 | 2714.61385 |
| Incidence | China | Male | 25-29 years | Vitamin A deficiency | Rate | 2021 | 2106.526835 |
| Incidence | China | Female | 25-29 years | Vitamin A deficiency | Rate | 2021 | 2578.224574 |
| Incidence | China | Male | 30-34 years | Vitamin A deficiency | Rate | 2021 | 2023.621117 |
| Incidence | China | Female | 30-34 years | Vitamin A deficiency | Rate | 2021 | 2380.21812 |
| Incidence | China | Male | 35-39 years | Vitamin A deficiency | Rate | 2021 | 1811.467488 |
| Incidence | China | Female | 35-39 years | Vitamin A deficiency | Rate | 2021 | 1942.729726 |
| Incidence | China | Male | 40-44 years | Vitamin A deficiency | Rate | 2021 | 1503.292947 |
| Incidence | China | Female | 40-44 years | Vitamin A deficiency | Rate | 2021 | 1611.800709 |
| Incidence | China | Male | 45-49 years | Vitamin A deficiency | Rate | 2021 | 1221.623495 |
| Incidence | China | Female | 45-49 years | Vitamin A deficiency | Rate | 2021 | 1301.403085 |
| Incidence | China | Male | 50-54 years | Vitamin A deficiency | Rate | 2021 | 866.7569232 |
| Incidence | China | Female | 50-54 years | Vitamin A deficiency | Rate | 2021 | 1022.843776 |
| Incidence | China | Male | 55-59 years | Vitamin A deficiency | Rate | 2021 | 584.6650194 |
| Incidence | China | Female | 55-59 years | Vitamin A deficiency | Rate | 2021 | 778.465237 |
| Incidence | China | Male | 5-9 years | Vitamin A deficiency | Rate | 2021 | 2657.643291 |
| Incidence | China | Female | 5-9 years | Vitamin A deficiency | Rate | 2021 | 2944.936905 |
| Incidence | China | Male | 60-64 years | Vitamin A deficiency | Rate | 2021 | 410.4880021 |
| Incidence | China | Female | 60-64 years | Vitamin A deficiency | Rate | 2021 | 594.6696458 |
| Incidence | China | Male | 65-69 years | Vitamin A deficiency | Rate | 2021 | 289.6682744 |
| Incidence | China | Female | 65-69 years | Vitamin A deficiency | Rate | 2021 | 450.4444589 |
| Incidence | China | Male | 70-74 years | Vitamin A deficiency | Rate | 2021 | 235.639422 |
| Incidence | China | Female | 70-74 years | Vitamin A deficiency | Rate | 2021 | 360.4853117 |
| Incidence | China | Male | 75-79 years | Vitamin A deficiency | Rate | 2021 | 174.5268504 |
| Incidence | China | Female | 75-79 years | Vitamin A deficiency | Rate | 2021 | 280.3876179 |
| Incidence | China | Male | 80-84 years | Vitamin A deficiency | Rate | 2021 | 171.7326654 |
| Incidence | China | Female | 80-84 years | Vitamin A deficiency | Rate | 2021 | 284.568355 |
| Incidence | China | Male | 85-89 years | Vitamin A deficiency | Rate | 2021 | 175.9070295 |
| Incidence | China | Female | 85-89 years | Vitamin A deficiency | Rate | 2021 | 280.090329 |
| Incidence | China | Male | 90-94 years | Vitamin A deficiency | Rate | 2021 | 178.186916 |
| Incidence | China | Female | 90-94 years | Vitamin A deficiency | Rate | 2021 | 286.3477504 |
| Incidence | China | Male | 95+ years | Vitamin A deficiency | Rate | 2021 | 177.3362599 |
| Incidence | China | Female | 95+ years | Vitamin A deficiency | Rate | 2021 | 310.5031199 |

| Age and gender differences in the Prevalence of vitamin A deficiency in China from 1990 to 2021 | | | | | | | |
| --- | --- | --- | --- | --- | --- | --- | --- |
| Measure | Location | Sex | Age | Cause | Metric | Year | Value |
| Prevalence | China | Male | <5 years | Vitamin A deficiency | Rate | 2021 | 2786.162951 |
| Prevalence | China | Female | <5 years | Vitamin A deficiency | Rate | 2021 | 3168.639994 |
| Prevalence | China | Male | 10-14 years | Vitamin A deficiency | Rate | 2021 | 2568.068301 |
| Prevalence | China | Female | 10-14 years | Vitamin A deficiency | Rate | 2021 | 2820.03416 |
| Prevalence | China | Male | 15-19 years | Vitamin A deficiency | Rate | 2021 | 2180.931243 |
| Prevalence | China | Female | 15-19 years | Vitamin A deficiency | Rate | 2021 | 2558.144787 |
| Prevalence | China | Male | 20-24 years | Vitamin A deficiency | Rate | 2021 | 2218.051657 |
| Prevalence | China | Female | 20-24 years | Vitamin A deficiency | Rate | 2021 | 2711.671359 |
| Prevalence | China | Male | 25-29 years | Vitamin A deficiency | Rate | 2021 | 2102.088417 |
| Prevalence | China | Female | 25-29 years | Vitamin A deficiency | Rate | 2021 | 2575.899713 |
| Prevalence | China | Male | 30-34 years | Vitamin A deficiency | Rate | 2021 | 2019.946912 |
| Prevalence | China | Female | 30-34 years | Vitamin A deficiency | Rate | 2021 | 2378.376328 |
| Prevalence | China | Male | 35-39 years | Vitamin A deficiency | Rate | 2021 | 1808.600533 |
| Prevalence | China | Female | 35-39 years | Vitamin A deficiency | Rate | 2021 | 1941.350239 |
| Prevalence | China | Male | 40-44 years | Vitamin A deficiency | Rate | 2021 | 1501.32495 |
| Prevalence | China | Female | 40-44 years | Vitamin A deficiency | Rate | 2021 | 1610.813242 |
| Prevalence | China | Male | 45-49 years | Vitamin A deficiency | Rate | 2021 | 1220.497719 |
| Prevalence | China | Female | 45-49 years | Vitamin A deficiency | Rate | 2021 | 1300.762368 |
| Prevalence | China | Male | 50-54 years | Vitamin A deficiency | Rate | 2021 | 866.1409902 |
| Prevalence | China | Female | 50-54 years | Vitamin A deficiency | Rate | 2021 | 1022.460531 |
| Prevalence | China | Male | 55-59 years | Vitamin A deficiency | Rate | 2021 | 584.3612602 |
| Prevalence | China | Female | 55-59 years | Vitamin A deficiency | Rate | 2021 | 778.261059 |
| Prevalence | China | Male | 5-9 years | Vitamin A deficiency | Rate | 2021 | 2649.469163 |
| Prevalence | China | Female | 5-9 years | Vitamin A deficiency | Rate | 2021 | 2940.648992 |
| Prevalence | China | Male | 60-64 years | Vitamin A deficiency | Rate | 2021 | 410.4141047 |
| Prevalence | China | Female | 60-64 years | Vitamin A deficiency | Rate | 2021 | 594.5672849 |
| Prevalence | China | Male | 65-69 years | Vitamin A deficiency | Rate | 2021 | 289.7823148 |
| Prevalence | China | Female | 65-69 years | Vitamin A deficiency | Rate | 2021 | 450.4068618 |
| Prevalence | China | Male | 70-74 years | Vitamin A deficiency | Rate | 2021 | 235.8950798 |
| Prevalence | China | Female | 70-74 years | Vitamin A deficiency | Rate | 2021 | 360.4966888 |
| Prevalence | China | Male | 75-79 years | Vitamin A deficiency | Rate | 2021 | 174.9017569 |
| Prevalence | China | Female | 75-79 years | Vitamin A deficiency | Rate | 2021 | 280.4148081 |
| Prevalence | China | Male | 80-84 years | Vitamin A deficiency | Rate | 2021 | 172.0574764 |
| Prevalence | China | Female | 80-84 years | Vitamin A deficiency | Rate | 2021 | 284.5896371 |
| Prevalence | China | Male | 85-89 years | Vitamin A deficiency | Rate | 2021 | 176.1431101 |
| Prevalence | China | Female | 85-89 years | Vitamin A deficiency | Rate | 2021 | 280.1024235 |
| Prevalence | China | Male | 90-94 years | Vitamin A deficiency | Rate | 2021 | 178.3639645 |
| Prevalence | China | Female | 90-94 years | Vitamin A deficiency | Rate | 2021 | 286.3538446 |
| Prevalence | China | Male | 95+ years | Vitamin A deficiency | Rate | 2021 | 177.4933074 |
| Prevalence | China | Female | 95+ years | Vitamin A deficiency | Rate | 2021 | 310.503652 |

| Age and gender differences in the DALYs of vitamin A deficiency in China from 1990 to 2021 | | | | | | | |
| --- | --- | --- | --- | --- | --- | --- | --- |
| Measure | Location | Sex | Age | Cause | Metric | Year | Value |
| DALYs | China | Male | <5 years | Vitamin A deficiency | Rate | 2021 | 11.69726405 |
| DALYs | China | Female | <5 years | Vitamin A deficiency | Rate | 2021 | 8.970289919 |
| DALYs | China | Male | 5-9 years | Vitamin A deficiency | Rate | 2021 | 6.894690764 |
| DALYs | China | Female | 5-9 years | Vitamin A deficiency | Rate | 2021 | 6.095660879 |
| DALYs | China | Male | 10-14 years | Vitamin A deficiency | Rate | 2021 | 4.560205876 |
| DALYs | China | Female | 10-14 years | Vitamin A deficiency | Rate | 2021 | 4.457615337 |
| DALYs | China | Male | 15-19 years | Vitamin A deficiency | Rate | 2021 | 3.114646292 |
| DALYs | China | Female | 15-19 years | Vitamin A deficiency | Rate | 2021 | 2.167651709 |
| DALYs | China | Male | 20-24 years | Vitamin A deficiency | Rate | 2021 | 2.600266075 |
| DALYs | China | Female | 20-24 years | Vitamin A deficiency | Rate | 2021 | 1.777858588 |
| DALYs | China | Male | 25-29 years | Vitamin A deficiency | Rate | 2021 | 2.36036369 |
| DALYs | China | Female | 25-29 years | Vitamin A deficiency | Rate | 2021 | 1.616781377 |
| DALYs | China | Male | 30-34 years | Vitamin A deficiency | Rate | 2021 | 2.189992968 |
| DALYs | China | Female | 30-34 years | Vitamin A deficiency | Rate | 2021 | 1.516798138 |
| DALYs | China | Male | 35-39 years | Vitamin A deficiency | Rate | 2021 | 2.025745584 |
| DALYs | China | Female | 35-39 years | Vitamin A deficiency | Rate | 2021 | 1.428200055 |
| DALYs | China | Male | 40-44 years | Vitamin A deficiency | Rate | 2021 | 1.883387116 |
| DALYs | China | Female | 40-44 years | Vitamin A deficiency | Rate | 2021 | 1.425709813 |
| DALYs | China | Male | 45-49 years | Vitamin A deficiency | Rate | 2021 | 1.735306144 |
| DALYs | China | Female | 45-49 years | Vitamin A deficiency | Rate | 2021 | 1.547419034 |
| DALYs | China | Male | 50-54 years | Vitamin A deficiency | Rate | 2021 | 1.689506656 |
| DALYs | China | Female | 50-54 years | Vitamin A deficiency | Rate | 2021 | 1.567648543 |
| DALYs | China | Male | 55-59 years | Vitamin A deficiency | Rate | 2021 | 1.666632545 |
| DALYs | China | Female | 55-59 years | Vitamin A deficiency | Rate | 2021 | 1.558781879 |
| DALYs | China | Male | 60-64 years | Vitamin A deficiency | Rate | 2021 | 1.658590086 |
| DALYs | China | Female | 60-64 years | Vitamin A deficiency | Rate | 2021 | 1.554786699 |
| DALYs | China | Male | 65-69 years | Vitamin A deficiency | Rate | 2021 | 1.700787215 |
| DALYs | China | Female | 65-69 years | Vitamin A deficiency | Rate | 2021 | 1.640105514 |
| DALYs | China | Male | 70-74 years | Vitamin A deficiency | Rate | 2021 | 1.730849292 |
| DALYs | China | Female | 70-74 years | Vitamin A deficiency | Rate | 2021 | 1.68382066 |
| DALYs | China | Male | 75-79 years | Vitamin A deficiency | Rate | 2021 | 1.628131771 |
| DALYs | China | Female | 75-79 years | Vitamin A deficiency | Rate | 2021 | 1.567596736 |
| DALYs | China | Male | 80-84 years | Vitamin A deficiency | Rate | 2021 | 1.453976496 |
| DALYs | China | Female | 80-84 years | Vitamin A deficiency | Rate | 2021 | 1.407831782 |
| DALYs | China | Male | 85-89 years | Vitamin A deficiency | Rate | 2021 | 1.263346337 |
| DALYs | China | Female | 85-89 years | Vitamin A deficiency | Rate | 2021 | 1.241890004 |
| DALYs | China | Male | 90-94 years | Vitamin A deficiency | Rate | 2021 | 0.960410829 |
| DALYs | China | Female | 90-94 years | Vitamin A deficiency | Rate | 2021 | 1.03722543 |
| DALYs | China | Male | 95+ years | Vitamin A deficiency | Rate | 2021 | 0.743129026 |
| DALYs | China | Female | 95+ years | Vitamin A deficiency | Rate | 2021 | 0.933988659 |

| Annual percentage change in the Incidence of vitamin A deficiency in China from 1990 to 2021 | | | | | |
| --- | --- | --- | --- | --- | --- |
| Age group | APC（95% CI） | APC（95% CI） | APC（95% CI） | APC（95% CI） | P-value |
| 1990-1995 | -4.326515989 | -4.904258822 | -3.74526315 | -4.33 (-4.90 - -3.75) | 0 |
| 1995-2004 | -5.561938477 | -5.87034071 | -5.252525809 | -5.56 (-5.87 - -5.25) | 0 |
| 2004-2008 | -7.821751524 | -8.86032809 | -6.771246693 | -7.82 (-8.86 - -6.77) | 0 |
| 2008-2021 | -6.043943776 | -6.220227667 | -5.86723438 | -6.04 (-6.22 - -5.87) | 0 |

| Annual percentage change in the Prevalence of vitamin A deficiency in China from 1990 to 2021 | | | | | |
| --- | --- | --- | --- | --- | --- |
| Age group | APC（95% CI） | APC（95% CI） | APC（95% CI） | APC（95% CI） | P-value |
| 1990-2002 | -5.034925612 | -5.181059274 | -4.888471618 | -5.035 (-5.181 - -4.888) | 0 |
| 2002-2010 | -7.40499616 | -7.701380384 | -7.107660203 | -7.405 (-7.701 - -7.108) | 0 |
| 2010-2016 | -5.233953484 | -5.70348938 | -4.762174841 | -5.234 (-5.703 - -4.762) | 0 |
| 2016-2021 | -7.318008617 | -7.924933369 | -6.706989948 | -7.318 (-7.925 - -6.707) | 0 |

| Annual percentage change in the DALYs of vitamin A deficiency in China from 1990 to 2021 | | | | | |
| --- | --- | --- | --- | --- | --- |
| Age group | APC（95% CI） | APC（95% CI） | APC（95% CI） | APC（95% CI） | P-value |
| 1990-1992 | -5.510644685 | -6.704844184 | -4.301159156 | -5.511 (-6.705 - -4.301) | 0 |
| 1992-2005 | -7.266463101 | -7.390087164 | -7.142766871 | -7.266 (-7.390 - -7.143) | 0 |
| 2005-2012 | -3.362275671 | -3.690189691 | -3.033148209 | -3.362 (-3.690 - -3.033) | 0 |
| 2012-2021 | 0.510347861 | 0.277213528 | 0.744014133 | 0.510 (0.277 - 0.744) | 0 |

| A breakdown analysis of the Incidence of vitamin A deficiency in China from 1990 to 2021 | | | | | | | | |
| --- | --- | --- | --- | --- | --- | --- | --- | --- |
| Location | Sex | Overall-Difference | Aging | Population | Epidemiological-Change | Aging-Percentage | Population-Percentage | Epidemiological-Change-Percentage |
| China | Both | -104423293.3 | -18074442.07 | 14551955.6 | -100900806.8 | 17.31 | -13.94 | 96.63 |

| A breakdown analysis of the Prevalence of vitamin A deficiency in China from 1990 to 2021 | | | | | | | | |
| --- | --- | --- | --- | --- | --- | --- | --- | --- |
| Location | Sex | Overall-Difference | Aging | Population | Epidemiological-Change | Aging-Percentage | Population-Percentage | Epidemiological-Change-Percentage |
| China | Both | -104435878.4 | -18062534.66 | 14546146.86 | -100919490.6 | 17.3 | -13.93 | 96.63 |

| A breakdown analysis of the DALYs of vitamin A deficiency in China from 1990 to 2021 | | | | | | | | |
| --- | --- | --- | --- | --- | --- | --- | --- | --- |
| Location | Sex | Overall-Difference | Aging | Population | Epidemiological-Change | Aging-Percentage | Population-Percentage | Epidemiological-Change-Percentage |
| China | Both | -80466.4 | -23083.62 | 14938.26 | -72321.04 | 28.69 | -18.56 | 89.88 |

| Predicted disease burden of vitamin A deficiency in China, 1990-2035 | | |
| --- | --- | --- |
| Time | Group | Value |
| 1990 | <5 years | 21209.29 |
| 1991 | <5 years | 20082.78 |
| 1992 | <5 years | 18953.38 |
| 1993 | <5 years | 17838.03 |
| 1994 | <5 years | 16748.95 |
| 1995 | <5 years | 15686.22 |
| 1996 | <5 years | 14593.20 |
| 1997 | <5 years | 13455.40 |
| 1998 | <5 years | 12344.65 |
| 1999 | <5 years | 11336.33 |
| 2000 | <5 years | 10499.14 |
| 2001 | <5 years | 9816.82 |
| 2002 | <5 years | 9205.36 |
| 2003 | <5 years | 8635.80 |
| 2004 | <5 years | 8098.52 |
| 2005 | <5 years | 7586.93 |
| 2006 | <5 years | 7074.83 |
| 2007 | <5 years | 6559.97 |
| 2008 | <5 years | 6069.32 |
| 2009 | <5 years | 5632.91 |
| 2010 | <5 years | 5277.72 |
| 2011 | <5 years | 5008.02 |
| 2012 | <5 years | 4811.81 |
| 2013 | <5 years | 4649.49 |
| 2014 | <5 years | 4476.84 |
| 2015 | <5 years | 4286.31 |
| 2016 | <5 years | 4074.66 |
| 2017 | <5 years | 3845.35 |
| 2018 | <5 years | 3607.38 |
| 2019 | <5 years | 3376.98 |
| 2020 | <5 years | 3151.90 |
| 2021 | <5 years | 2972.56 |
| 2022 | <5 years | 2786.31 |
| 2023 | <5 years | 2630.79 |
| 2024 | <5 years | 2484.11 |
| 2025 | <5 years | 2345.78 |
| 2026 | <5 years | 2215.34 |
| 2027 | <5 years | 2092.35 |
| 2028 | <5 years | 1976.38 |
| 2029 | <5 years | 1867.04 |
| 2030 | <5 years | 1763.97 |
| 2031 | <5 years | 1666.79 |
| 2032 | <5 years | 1575.18 |
| 2033 | <5 years | 1488.83 |
| 2034 | <5 years | 1407.43 |
| 2035 | <5 years | 1330.69 |
| 1990 | 5-9 years | 14587.09 |
| 1991 | 5-9 years | 14147.26 |
| 1992 | 5-9 years | 13717.72 |
| 1993 | 5-9 years | 13293.26 |
| 1994 | 5-9 years | 12871.26 |
| 1995 | 5-9 years | 12452.02 |
| 1996 | 5-9 years | 12021.47 |
| 1997 | 5-9 years | 11573.96 |
| 1998 | 5-9 years | 11124.08 |
| 1999 | 5-9 years | 10687.31 |
| 2000 | 5-9 years | 10279.45 |
| 2001 | 5-9 years | 9905.52 |
| 2002 | 5-9 years | 9546.45 |
| 2003 | 5-9 years | 9183.81 |
| 2004 | 5-9 years | 8799.54 |
| 2005 | 5-9 years | 8377.31 |
| 2006 | 5-9 years | 7878.18 |
| 2007 | 5-9 years | 7311.05 |
| 2008 | 5-9 years | 6731.44 |
| 2009 | 5-9 years | 6195.02 |
| 2010 | 5-9 years | 5757.04 |
| 2011 | 5-9 years | 5414.49 |
| 2012 | 5-9 years | 5117.27 |
| 2013 | 5-9 years | 4848.64 |
| 2014 | 5-9 years | 4591.43 |
| 2015 | 5-9 years | 4328.31 |
| 2016 | 5-9 years | 4055.69 |
| 2017 | 5-9 years | 3783.28 |
| 2018 | 5-9 years | 3514.53 |
| 2019 | 5-9 years | 3253.04 |
| 2020 | 5-9 years | 2997.54 |
| 2021 | 5-9 years | 2792.12 |
| 2022 | 5-9 years | 2647.90 |
| 2023 | 5-9 years | 2498.97 |
| 2024 | 5-9 years | 2358.86 |
| 2025 | 5-9 years | 2226.87 |
| 2026 | 5-9 years | 2102.42 |
| 2027 | 5-9 years | 1985.02 |
| 2028 | 5-9 years | 1874.28 |
| 2029 | 5-9 years | 1769.83 |
| 2030 | 5-9 years | 1671.33 |
| 2031 | 5-9 years | 1578.44 |
| 2032 | 5-9 years | 1490.85 |
| 2033 | 5-9 years | 1408.27 |
| 2034 | 5-9 years | 1330.40 |
| 2035 | 5-9 years | 1256.99 |
| 1990 | 10-14 years | 14470.23 |
| 1991 | 10-14 years | 14087.73 |
| 1992 | 10-14 years | 13684.77 |
| 1993 | 10-14 years | 13276.62 |
| 1994 | 10-14 years | 12873.75 |
| 1995 | 10-14 years | 12479.79 |
| 1996 | 10-14 years | 12056.32 |
| 1997 | 10-14 years | 11583.65 |
| 1998 | 10-14 years | 11086.58 |
| 1999 | 10-14 years | 10591.63 |
| 2000 | 10-14 years | 10127.03 |
| 2001 | 10-14 years | 9689.68 |
| 2002 | 10-14 years | 9254.92 |
| 2003 | 10-14 years | 8818.77 |
| 2004 | 10-14 years | 8377.56 |
| 2005 | 10-14 years | 7927.00 |
| 2006 | 10-14 years | 7429.84 |
| 2007 | 10-14 years | 6882.24 |
| 2008 | 10-14 years | 6331.86 |
| 2009 | 10-14 years | 5828.31 |
| 2010 | 10-14 years | 5423.18 |
| 2011 | 10-14 years | 5110.96 |
| 2012 | 10-14 years | 4841.27 |
| 2013 | 10-14 years | 4597.40 |
| 2014 | 10-14 years | 4362.35 |
| 2015 | 10-14 years | 4119.02 |
| 2016 | 10-14 years | 3865.79 |
| 2017 | 10-14 years | 3614.43 |
| 2018 | 10-14 years | 3366.70 |
| 2019 | 10-14 years | 3123.90 |
| 2020 | 10-14 years | 2883.78 |
| 2021 | 10-14 years | 2690.83 |
| 2022 | 10-14 years | 2570.64 |
| 2023 | 10-14 years | 2420.29 |
| 2024 | 10-14 years | 2280.34 |
| 2025 | 10-14 years | 2149.72 |
| 2026 | 10-14 years | 2027.56 |
| 2027 | 10-14 years | 1913.05 |
| 2028 | 10-14 years | 1805.50 |
| 2029 | 10-14 years | 1704.30 |
| 2030 | 10-14 years | 1608.97 |
| 2031 | 10-14 years | 1519.08 |
| 2032 | 10-14 years | 1434.29 |
| 2033 | 10-14 years | 1354.30 |
| 2034 | 10-14 years | 1278.86 |
| 2035 | 10-14 years | 1207.71 |
| 1990 | 15-19 years | 11747.41 |
| 1991 | 15-19 years | 11373.99 |
| 1992 | 15-19 years | 10969.65 |
| 1993 | 15-19 years | 10537.49 |
| 1994 | 15-19 years | 10091.51 |
| 1995 | 15-19 years | 9645.78 |
| 1996 | 15-19 years | 9193.07 |
| 1997 | 15-19 years | 8732.39 |
| 1998 | 15-19 years | 8285.55 |
| 1999 | 15-19 years | 7870.15 |
| 2000 | 15-19 years | 7498.29 |
| 2001 | 15-19 years | 7162.13 |
| 2002 | 15-19 years | 6842.70 |
| 2003 | 15-19 years | 6531.63 |
| 2004 | 15-19 years | 6221.57 |
| 2005 | 15-19 years | 5905.68 |
| 2006 | 15-19 years | 5560.59 |
| 2007 | 15-19 years | 5188.65 |
| 2008 | 15-19 years | 4821.73 |
| 2009 | 15-19 years | 4492.02 |
| 2010 | 15-19 years | 4231.61 |
| 2011 | 15-19 years | 4036.28 |
| 2012 | 15-19 years | 3870.25 |
| 2013 | 15-19 years | 3719.44 |
| 2014 | 15-19 years | 3570.08 |
| 2015 | 15-19 years | 3408.95 |
| 2016 | 15-19 years | 3234.25 |
| 2017 | 15-19 years | 3054.45 |
| 2018 | 15-19 years | 2872.18 |
| 2019 | 15-19 years | 2690.42 |
| 2020 | 15-19 years | 2506.97 |
| 2021 | 15-19 years | 2360.57 |
| 2022 | 15-19 years | 2108.03 |
| 2023 | 15-19 years | 1975.88 |
| 2024 | 15-19 years | 1853.37 |
| 2025 | 15-19 years | 1740.05 |
| 2026 | 15-19 years | 1635.31 |
| 2027 | 15-19 years | 1538.38 |
| 2028 | 15-19 years | 1448.43 |
| 2029 | 15-19 years | 1364.71 |
| 2030 | 15-19 years | 1286.57 |
| 2031 | 15-19 years | 1213.48 |
| 2032 | 15-19 years | 1144.97 |
| 2033 | 15-19 years | 1080.62 |
| 2034 | 15-19 years | 1020.08 |
| 2035 | 15-19 years | 963.04 |
| 1990 | 20-24 years | 12138.43 |
| 1991 | 20-24 years | 11797.14 |
| 1992 | 20-24 years | 11437.11 |
| 1993 | 20-24 years | 11074.32 |
| 1994 | 20-24 years | 10710.38 |
| 1995 | 20-24 years | 10348.81 |
| 1996 | 20-24 years | 9967.32 |
| 1997 | 20-24 years | 9548.39 |
| 1998 | 20-24 years | 9106.26 |
| 1999 | 20-24 years | 8665.56 |
| 2000 | 20-24 years | 8251.86 |
| 2001 | 20-24 years | 7865.96 |
| 2002 | 20-24 years | 7490.31 |
| 2003 | 20-24 years | 7125.06 |
| 2004 | 20-24 years | 6768.64 |
| 2005 | 20-24 years | 6416.27 |
| 2006 | 20-24 years | 6032.47 |
| 2007 | 20-24 years | 5611.68 |
| 2008 | 20-24 years | 5191.52 |
| 2009 | 20-24 years | 4810.82 |
| 2010 | 20-24 years | 4509.49 |
| 2011 | 20-24 years | 4284.43 |
| 2012 | 20-24 years | 4095.01 |
| 2013 | 20-24 years | 3925.99 |
| 2014 | 20-24 years | 3762.29 |
| 2015 | 20-24 years | 3588.65 |
| 2016 | 20-24 years | 3402.07 |
| 2017 | 20-24 years | 3210.16 |
| 2018 | 20-24 years | 3014.48 |
| 2019 | 20-24 years | 2817.47 |
| 2020 | 20-24 years | 2616.90 |
| 2021 | 20-24 years | 2453.73 |
| 2022 | 20-24 years | 2357.00 |
| 2023 | 20-24 years | 2211.56 |
| 2024 | 20-24 years | 2073.55 |
| 2025 | 20-24 years | 1943.14 |
| 2026 | 20-24 years | 1820.47 |
| 2027 | 20-24 years | 1705.61 |
| 2028 | 20-24 years | 1598.72 |
| 2029 | 20-24 years | 1499.63 |
| 2030 | 20-24 years | 1407.97 |
| 2031 | 20-24 years | 1323.24 |
| 2032 | 20-24 years | 1244.84 |
| 2033 | 20-24 years | 1172.08 |
| 2034 | 20-24 years | 1104.36 |
| 2035 | 20-24 years | 1041.15 |
| 1990 | 25-29 years | 11316.20 |
| 1991 | 25-29 years | 11069.01 |
| 1992 | 25-29 years | 10790.02 |
| 1993 | 25-29 years | 10482.10 |
| 1994 | 25-29 years | 10153.40 |
| 1995 | 25-29 years | 9812.90 |
| 1996 | 25-29 years | 9441.34 |
| 1997 | 25-29 years | 9037.74 |
| 1998 | 25-29 years | 8636.47 |
| 1999 | 25-29 years | 8256.80 |
| 2000 | 25-29 years | 7917.85 |
| 2001 | 25-29 years | 7605.09 |
| 2002 | 25-29 years | 7282.84 |
| 2003 | 25-29 years | 6942.55 |
| 2004 | 25-29 years | 6586.34 |
| 2005 | 25-29 years | 6218.74 |
| 2006 | 25-29 years | 5824.83 |
| 2007 | 25-29 years | 5409.29 |
| 2008 | 25-29 years | 5006.06 |
| 2009 | 25-29 years | 4646.24 |
| 2010 | 25-29 years | 4358.00 |
| 2011 | 25-29 years | 4135.44 |
| 2012 | 25-29 years | 3946.27 |
| 2013 | 25-29 years | 3775.94 |
| 2014 | 25-29 years | 3610.57 |
| 2015 | 25-29 years | 3437.04 |
| 2016 | 25-29 years | 3252.40 |
| 2017 | 25-29 years | 3063.25 |
| 2018 | 25-29 years | 2871.42 |
| 2019 | 25-29 years | 2679.67 |
| 2020 | 25-29 years | 2486.17 |
| 2021 | 25-29 years | 2329.47 |
| 2022 | 25-29 years | 2357.04 |
| 2023 | 25-29 years | 2224.69 |
| 2024 | 25-29 years | 2096.71 |
| 2025 | 25-29 years | 1973.47 |
| 2026 | 25-29 years | 1855.33 |
| 2027 | 25-29 years | 1742.48 |
| 2028 | 25-29 years | 1634.99 |
| 2029 | 25-29 years | 1532.99 |
| 2030 | 25-29 years | 1436.62 |
| 2031 | 25-29 years | 1345.95 |
| 2032 | 25-29 years | 1261.06 |
| 2033 | 25-29 years | 1182.05 |
| 2034 | 25-29 years | 1108.81 |
| 2035 | 25-29 years | 1041.07 |
| 1990 | 30-34 years | 10034.50 |
| 1991 | 30-34 years | 9713.92 |
| 1992 | 30-34 years | 9450.45 |
| 1993 | 30-34 years | 9225.05 |
| 1994 | 30-34 years | 9001.70 |
| 1995 | 30-34 years | 8761.10 |
| 1996 | 30-34 years | 8490.46 |
| 1997 | 30-34 years | 8194.98 |
| 1998 | 30-34 years | 7888.33 |
| 1999 | 30-34 years | 7587.66 |
| 2000 | 30-34 years | 7310.33 |
| 2001 | 30-34 years | 7032.81 |
| 2002 | 30-34 years | 6732.36 |
| 2003 | 30-34 years | 6424.04 |
| 2004 | 30-34 years | 6111.11 |
| 2005 | 30-34 years | 5797.95 |
| 2006 | 30-34 years | 5460.53 |
| 2007 | 30-34 years | 5088.36 |
| 2008 | 30-34 years | 4710.37 |
| 2009 | 30-34 years | 4362.14 |
| 2010 | 30-34 years | 4078.42 |
| 2011 | 30-34 years | 3861.63 |
| 2012 | 30-34 years | 3682.03 |
| 2013 | 30-34 years | 3525.89 |
| 2014 | 30-34 years | 3378.89 |
| 2015 | 30-34 years | 3225.46 |
| 2016 | 30-34 years | 3057.52 |
| 2017 | 30-34 years | 2881.65 |
| 2018 | 30-34 years | 2702.09 |
| 2019 | 30-34 years | 2523.49 |
| 2020 | 30-34 years | 2342.38 |
| 2021 | 30-34 years | 2195.74 |
| 2022 | 30-34 years | 2196.28 |
| 2023 | 30-34 years | 2087.53 |
| 2024 | 30-34 years | 1981.50 |
| 2025 | 30-34 years | 1878.27 |
| 2026 | 30-34 years | 1778.00 |
| 2027 | 30-34 years | 1680.75 |
| 2028 | 30-34 years | 1586.41 |
| 2029 | 30-34 years | 1495.18 |
| 2030 | 30-34 years | 1407.33 |
| 2031 | 30-34 years | 1323.11 |
| 2032 | 30-34 years | 1242.66 |
| 2033 | 30-34 years | 1166.03 |
| 2034 | 30-34 years | 1093.31 |
| 2035 | 30-34 years | 1024.60 |
| 1990 | 35-39 years | 8492.13 |
| 1991 | 35-39 years | 8307.82 |
| 1992 | 35-39 years | 8082.37 |
| 1993 | 35-39 years | 7815.20 |
| 1994 | 35-39 years | 7520.35 |
| 1995 | 35-39 years | 7216.79 |
| 1996 | 35-39 years | 6913.96 |
| 1997 | 35-39 years | 6624.38 |
| 1998 | 35-39 years | 6354.87 |
| 1999 | 35-39 years | 6104.04 |
| 2000 | 35-39 years | 5883.74 |
| 2001 | 35-39 years | 5673.68 |
| 2002 | 35-39 years | 5445.11 |
| 2003 | 35-39 years | 5200.47 |
| 2004 | 35-39 years | 4944.53 |
| 2005 | 35-39 years | 4682.46 |
| 2006 | 35-39 years | 4396.26 |
| 2007 | 35-39 years | 4086.78 |
| 2008 | 35-39 years | 3786.88 |
| 2009 | 35-39 years | 3522.21 |
| 2010 | 35-39 years | 3319.96 |
| 2011 | 35-39 years | 3173.73 |
| 2012 | 35-39 years | 3048.73 |
| 2013 | 35-39 years | 2931.57 |
| 2014 | 35-39 years | 2813.02 |
| 2015 | 35-39 years | 2684.56 |
| 2016 | 35-39 years | 2547.48 |
| 2017 | 35-39 years | 2409.43 |
| 2018 | 35-39 years | 2271.89 |
| 2019 | 35-39 years | 2135.62 |
| 2020 | 35-39 years | 1991.71 |
| 2021 | 35-39 years | 1875.39 |
| 2022 | 35-39 years | 1794.80 |
| 2023 | 35-39 years | 1707.03 |
| 2024 | 35-39 years | 1625.21 |
| 2025 | 35-39 years | 1548.13 |
| 2026 | 35-39 years | 1474.35 |
| 2027 | 35-39 years | 1403.02 |
| 2028 | 35-39 years | 1333.57 |
| 2029 | 35-39 years | 1265.87 |
| 2030 | 35-39 years | 1199.95 |
| 2031 | 35-39 years | 1135.92 |
| 2032 | 35-39 years | 1073.81 |
| 2033 | 35-39 years | 1013.56 |
| 2034 | 35-39 years | 955.29 |
| 2035 | 35-39 years | 899.19 |
| 1990 | 40-44 years | 7308.19 |
| 1991 | 40-44 years | 7021.98 |
| 1992 | 40-44 years | 6752.63 |
| 1993 | 40-44 years | 6499.75 |
| 1994 | 40-44 years | 6260.93 |
| 1995 | 40-44 years | 6048.94 |
| 1996 | 40-44 years | 5830.56 |
| 1997 | 40-44 years | 5603.02 |
| 1998 | 40-44 years | 5369.14 |
| 1999 | 40-44 years | 5140.31 |
| 2000 | 40-44 years | 4930.31 |
| 2001 | 40-44 years | 4729.16 |
| 2002 | 40-44 years | 4525.41 |
| 2003 | 40-44 years | 4320.83 |
| 2004 | 40-44 years | 4112.05 |
| 2005 | 40-44 years | 3904.73 |
| 2006 | 40-44 years | 3679.52 |
| 2007 | 40-44 years | 3427.07 |
| 2008 | 40-44 years | 3171.53 |
| 2009 | 40-44 years | 2938.97 |
| 2010 | 40-44 years | 2755.46 |
| 2011 | 40-44 years | 2618.93 |
| 2012 | 40-44 years | 2506.04 |
| 2013 | 40-44 years | 2409.79 |
| 2014 | 40-44 years | 2318.46 |
| 2015 | 40-44 years | 2220.85 |
| 2016 | 40-44 years | 2114.00 |
| 2017 | 40-44 years | 2001.89 |
| 2018 | 40-44 years | 1885.32 |
| 2019 | 40-44 years | 1767.92 |
| 2020 | 40-44 years | 1650.13 |
| 2021 | 40-44 years | 1556.19 |
| 2022 | 40-44 years | 1512.45 |
| 2023 | 40-44 years | 1431.91 |
| 2024 | 40-44 years | 1355.99 |
| 2025 | 40-44 years | 1284.84 |
| 2026 | 40-44 years | 1218.82 |
| 2027 | 40-44 years | 1157.77 |
| 2028 | 40-44 years | 1101.18 |
| 2029 | 40-44 years | 1048.42 |
| 2030 | 40-44 years | 998.72 |
| 2031 | 40-44 years | 951.15 |
| 2032 | 40-44 years | 905.15 |
| 2033 | 40-44 years | 860.36 |
| 2034 | 40-44 years | 816.70 |
| 2035 | 40-44 years | 774.19 |
| 1990 | 45-49 years | 6006.48 |
| 1991 | 45-49 years | 5781.76 |
| 1992 | 45-49 years | 5555.65 |
| 1993 | 45-49 years | 5340.67 |
| 1994 | 45-49 years | 5134.86 |
| 1995 | 45-49 years | 4918.27 |
| 1996 | 45-49 years | 4699.64 |
| 1997 | 45-49 years | 4486.29 |
| 1998 | 45-49 years | 4286.18 |
| 1999 | 45-49 years | 4105.69 |
| 2000 | 45-49 years | 3959.34 |
| 2001 | 45-49 years | 3822.40 |
| 2002 | 45-49 years | 3675.72 |
| 2003 | 45-49 years | 3516.02 |
| 2004 | 45-49 years | 3346.06 |
| 2005 | 45-49 years | 3170.99 |
| 2006 | 45-49 years | 2977.16 |
| 2007 | 45-49 years | 2765.43 |
| 2008 | 45-49 years | 2556.73 |
| 2009 | 45-49 years | 2369.50 |
| 2010 | 45-49 years | 2228.95 |
| 2011 | 45-49 years | 2130.39 |
| 2012 | 45-49 years | 2045.50 |
| 2013 | 45-49 years | 1966.83 |
| 2014 | 45-49 years | 1887.87 |
| 2015 | 45-49 years | 1802.07 |
| 2016 | 45-49 years | 1709.70 |
| 2017 | 45-49 years | 1617.15 |
| 2018 | 45-49 years | 1525.80 |
| 2019 | 45-49 years | 1434.12 |
| 2020 | 45-49 years | 1337.72 |
| 2021 | 45-49 years | 1260.88 |
| 2022 | 45-49 years | 1247.06 |
| 2023 | 45-49 years | 1182.99 |
| 2024 | 45-49 years | 1121.09 |
| 2025 | 45-49 years | 1061.62 |
| 2026 | 45-49 years | 1005.05 |
| 2027 | 45-49 years | 951.48 |
| 2028 | 45-49 years | 900.83 |
| 2029 | 45-49 years | 853.09 |
| 2030 | 45-49 years | 808.34 |
| 2031 | 45-49 years | 766.83 |
| 2032 | 45-49 years | 728.43 |
| 2033 | 45-49 years | 692.84 |
| 2034 | 45-49 years | 659.66 |
| 2035 | 45-49 years | 628.40 |
| 1990 | 50-54 years | 4702.27 |
| 1991 | 50-54 years | 4509.82 |
| 1992 | 50-54 years | 4319.69 |
| 1993 | 50-54 years | 4133.23 |
| 1994 | 50-54 years | 3942.57 |
| 1995 | 50-54 years | 3755.61 |
| 1996 | 50-54 years | 3575.04 |
| 1997 | 50-54 years | 3399.84 |
| 1998 | 50-54 years | 3240.59 |
| 1999 | 50-54 years | 3097.72 |
| 2000 | 50-54 years | 2965.65 |
| 2001 | 50-54 years | 2846.70 |
| 2002 | 50-54 years | 2737.00 |
| 2003 | 50-54 years | 2631.30 |
| 2004 | 50-54 years | 2524.24 |
| 2005 | 50-54 years | 2415.00 |
| 2006 | 50-54 years | 2284.69 |
| 2007 | 50-54 years | 2132.24 |
| 2008 | 50-54 years | 1972.10 |
| 2009 | 50-54 years | 1821.80 |
| 2010 | 50-54 years | 1699.15 |
| 2011 | 50-54 years | 1607.56 |
| 2012 | 50-54 years | 1535.74 |
| 2013 | 50-54 years | 1476.14 |
| 2014 | 50-54 years | 1419.21 |
| 2015 | 50-54 years | 1358.47 |
| 2016 | 50-54 years | 1291.87 |
| 2017 | 50-54 years | 1221.35 |
| 2018 | 50-54 years | 1148.32 |
| 2019 | 50-54 years | 1075.06 |
| 2020 | 50-54 years | 1002.48 |
| 2021 | 50-54 years | 943.90 |
| 2022 | 50-54 years | 945.34 |
| 2023 | 50-54 years | 899.69 |
| 2024 | 50-54 years | 855.86 |
| 2025 | 50-54 years | 813.72 |
| 2026 | 50-54 years | 773.25 |
| 2027 | 50-54 years | 734.25 |
| 2028 | 50-54 years | 696.54 |
| 2029 | 50-54 years | 660.11 |
| 2030 | 50-54 years | 625.11 |
| 2031 | 50-54 years | 591.81 |
| 2032 | 50-54 years | 560.28 |
| 2033 | 50-54 years | 530.47 |
| 2034 | 50-54 years | 502.37 |
| 2035 | 50-54 years | 476.03 |
| 1990 | 55-59 years | 3548.14 |
| 1991 | 55-59 years | 3380.81 |
| 1992 | 55-59 years | 3219.94 |
| 1993 | 55-59 years | 3066.43 |
| 1994 | 55-59 years | 2922.49 |
| 1995 | 55-59 years | 2787.69 |
| 1996 | 55-59 years | 2650.59 |
| 1997 | 55-59 years | 2509.78 |
| 1998 | 55-59 years | 2372.16 |
| 1999 | 55-59 years | 2243.77 |
| 2000 | 55-59 years | 2131.93 |
| 2001 | 55-59 years | 2041.46 |
| 2002 | 55-59 years | 1962.55 |
| 2003 | 55-59 years | 1892.31 |
| 2004 | 55-59 years | 1821.72 |
| 2005 | 55-59 years | 1739.79 |
| 2006 | 55-59 years | 1637.50 |
| 2007 | 55-59 years | 1521.00 |
| 2008 | 55-59 years | 1404.01 |
| 2009 | 55-59 years | 1299.71 |
| 2010 | 55-59 years | 1223.41 |
| 2011 | 55-59 years | 1169.58 |
| 2012 | 55-59 years | 1123.36 |
| 2013 | 55-59 years | 1079.60 |
| 2014 | 55-59 years | 1034.96 |
| 2015 | 55-59 years | 986.96 |
| 2016 | 55-59 years | 935.78 |
| 2017 | 55-59 years | 884.49 |
| 2018 | 55-59 years | 833.28 |
| 2019 | 55-59 years | 781.38 |
| 2020 | 55-59 years | 725.95 |
| 2021 | 55-59 years | 681.74 |
| 2022 | 55-59 years | 679.51 |
| 2023 | 55-59 years | 647.23 |
| 2024 | 55-59 years | 616.36 |
| 2025 | 55-59 years | 586.85 |
| 2026 | 55-59 years | 558.73 |
| 2027 | 55-59 years | 531.92 |
| 2028 | 55-59 years | 506.25 |
| 2029 | 55-59 years | 481.59 |
| 2030 | 55-59 years | 457.89 |
| 2031 | 55-59 years | 435.13 |
| 2032 | 55-59 years | 413.19 |
| 2033 | 55-59 years | 391.98 |
| 2034 | 55-59 years | 371.49 |
| 2035 | 55-59 years | 351.80 |
| 1990 | 60-64 years | 2649.11 |
| 1991 | 60-64 years | 2524.18 |
| 1992 | 60-64 years | 2400.95 |
| 1993 | 60-64 years | 2279.08 |
| 1994 | 60-64 years | 2159.79 |
| 1995 | 60-64 years | 2043.59 |
| 1996 | 60-64 years | 1927.18 |
| 1997 | 60-64 years | 1809.27 |
| 1998 | 60-64 years | 1697.63 |
| 1999 | 60-64 years | 1600.03 |
| 2000 | 60-64 years | 1525.27 |
| 2001 | 60-64 years | 1469.15 |
| 2002 | 60-64 years | 1422.26 |
| 2003 | 60-64 years | 1377.89 |
| 2004 | 60-64 years | 1329.88 |
| 2005 | 60-64 years | 1270.98 |
| 2006 | 60-64 years | 1198.04 |
| 2007 | 60-64 years | 1114.81 |
| 2008 | 60-64 years | 1032.85 |
| 2009 | 60-64 years | 959.08 |
| 2010 | 60-64 years | 899.84 |
| 2011 | 60-64 years | 855.06 |
| 2012 | 60-64 years | 817.99 |
| 2013 | 60-64 years | 785.45 |
| 2014 | 60-64 years | 754.15 |
| 2015 | 60-64 years | 722.22 |
| 2016 | 60-64 years | 686.59 |
| 2017 | 60-64 years | 648.70 |
| 2018 | 60-64 years | 609.41 |
| 2019 | 60-64 years | 570.51 |
| 2020 | 60-64 years | 532.30 |
| 2021 | 60-64 years | 502.29 |
| 2022 | 60-64 years | 488.88 |
| 2023 | 60-64 years | 464.68 |
| 2024 | 60-64 years | 441.95 |
| 2025 | 60-64 years | 420.56 |
| 2026 | 60-64 years | 400.45 |
| 2027 | 60-64 years | 381.44 |
| 2028 | 60-64 years | 363.32 |
| 2029 | 60-64 years | 346.00 |
| 2030 | 60-64 years | 329.45 |
| 2031 | 60-64 years | 313.67 |
| 2032 | 60-64 years | 298.62 |
| 2033 | 60-64 years | 284.22 |
| 2034 | 60-64 years | 270.38 |
| 2035 | 60-64 years | 257.08 |
| 1990 | 65-69 years | 2099.43 |
| 1991 | 65-69 years | 1975.37 |
| 1992 | 65-69 years | 1855.96 |
| 1993 | 65-69 years | 1742.30 |
| 1994 | 65-69 years | 1634.85 |
| 1995 | 65-69 years | 1533.48 |
| 1996 | 65-69 years | 1434.12 |
| 1997 | 65-69 years | 1335.41 |
| 1998 | 65-69 years | 1242.17 |
| 1999 | 65-69 years | 1160.16 |
| 2000 | 65-69 years | 1094.53 |
| 2001 | 65-69 years | 1045.73 |
| 2002 | 65-69 years | 1006.25 |
| 2003 | 65-69 years | 971.77 |
| 2004 | 65-69 years | 937.78 |
| 2005 | 65-69 years | 900.70 |
| 2006 | 65-69 years | 852.71 |
| 2007 | 65-69 years | 796.13 |
| 2008 | 65-69 years | 737.27 |
| 2009 | 65-69 years | 683.68 |
| 2010 | 65-69 years | 641.42 |
| 2011 | 65-69 years | 611.22 |
| 2012 | 65-69 years | 585.94 |
| 2013 | 65-69 years | 564.78 |
| 2014 | 65-69 years | 544.51 |
| 2015 | 65-69 years | 521.86 |
| 2016 | 65-69 years | 496.87 |
| 2017 | 65-69 years | 471.63 |
| 2018 | 65-69 years | 446.15 |
| 2019 | 65-69 years | 420.45 |
| 2020 | 65-69 years | 393.54 |
| 2021 | 65-69 years | 371.34 |
| 2022 | 65-69 years | 354.61 |
| 2023 | 65-69 years | 336.66 |
| 2024 | 65-69 years | 319.48 |
| 2025 | 65-69 years | 303.13 |
| 2026 | 65-69 years | 287.75 |
| 2027 | 65-69 years | 273.33 |
| 2028 | 65-69 years | 259.80 |
| 2029 | 65-69 years | 247.10 |
| 2030 | 65-69 years | 235.15 |
| 2031 | 65-69 years | 223.91 |
| 2032 | 65-69 years | 213.28 |
| 2033 | 65-69 years | 203.16 |
| 2034 | 65-69 years | 193.48 |
| 2035 | 65-69 years | 184.22 |
| 1990 | 70-74 years | 1876.37 |
| 1991 | 70-74 years | 1758.31 |
| 1992 | 70-74 years | 1644.45 |
| 1993 | 70-74 years | 1536.21 |
| 1994 | 70-74 years | 1433.64 |
| 1995 | 70-74 years | 1337.60 |
| 1996 | 70-74 years | 1241.85 |
| 1997 | 70-74 years | 1146.39 |
| 1998 | 70-74 years | 1057.61 |
| 1999 | 70-74 years | 981.42 |
| 2000 | 70-74 years | 923.32 |
| 2001 | 70-74 years | 881.63 |
| 2002 | 70-74 years | 847.92 |
| 2003 | 70-74 years | 817.74 |
| 2004 | 70-74 years | 787.36 |
| 2005 | 70-74 years | 752.60 |
| 2006 | 70-74 years | 709.76 |
| 2007 | 70-74 years | 660.53 |
| 2008 | 70-74 years | 610.52 |
| 2009 | 70-74 years | 565.48 |
| 2010 | 70-74 years | 532.12 |
| 2011 | 70-74 years | 508.18 |
| 2012 | 70-74 years | 487.83 |
| 2013 | 70-74 years | 468.98 |
| 2014 | 70-74 years | 450.25 |
| 2015 | 70-74 years | 429.14 |
| 2016 | 70-74 years | 406.85 |
| 2017 | 70-74 years | 384.54 |
| 2018 | 70-74 years | 363.12 |
| 2019 | 70-74 years | 341.62 |
| 2020 | 70-74 years | 318.39 |
| 2021 | 70-74 years | 299.94 |
| 2022 | 70-74 years | 291.55 |
| 2023 | 70-74 years | 277.12 |
| 2024 | 70-74 years | 263.38 |
| 2025 | 70-74 years | 250.28 |
| 2026 | 70-74 years | 237.80 |
| 2027 | 70-74 years | 225.87 |
| 2028 | 70-74 years | 214.44 |
| 2029 | 70-74 years | 203.51 |
| 2030 | 70-74 years | 193.10 |
| 2031 | 70-74 years | 183.30 |
| 2032 | 70-74 years | 174.12 |
| 2033 | 70-74 years | 165.51 |
| 2034 | 70-74 years | 157.42 |
| 2035 | 70-74 years | 149.81 |
| 1990 | 75-79 years | 1568.09 |
| 1991 | 75-79 years | 1456.45 |
| 1992 | 75-79 years | 1348.81 |
| 1993 | 75-79 years | 1245.92 |
| 1994 | 75-79 years | 1149.02 |
| 1995 | 75-79 years | 1058.88 |
| 1996 | 75-79 years | 973.53 |
| 1997 | 75-79 years | 891.00 |
| 1998 | 75-79 years | 816.21 |
| 1999 | 75-79 years | 753.33 |
| 2000 | 75-79 years | 706.96 |
| 2001 | 75-79 years | 674.15 |
| 2002 | 75-79 years | 648.00 |
| 2003 | 75-79 years | 625.54 |
| 2004 | 75-79 years | 603.36 |
| 2005 | 75-79 years | 577.83 |
| 2006 | 75-79 years | 546.01 |
| 2007 | 75-79 years | 509.59 |
| 2008 | 75-79 years | 472.25 |
| 2009 | 75-79 years | 438.18 |
| 2010 | 75-79 years | 411.18 |
| 2011 | 75-79 years | 391.32 |
| 2012 | 75-79 years | 374.65 |
| 2013 | 75-79 years | 359.82 |
| 2014 | 75-79 years | 345.37 |
| 2015 | 75-79 years | 330.71 |
| 2016 | 75-79 years | 314.48 |
| 2017 | 75-79 years | 297.49 |
| 2018 | 75-79 years | 279.86 |
| 2019 | 75-79 years | 262.50 |
| 2020 | 75-79 years | 244.78 |
| 2021 | 75-79 years | 230.55 |
| 2022 | 75-79 years | 222.72 |
| 2023 | 75-79 years | 211.56 |
| 2024 | 75-79 years | 200.99 |
| 2025 | 75-79 years | 190.97 |
| 2026 | 75-79 years | 181.49 |
| 2027 | 75-79 years | 172.51 |
| 2028 | 75-79 years | 163.98 |
| 2029 | 75-79 years | 155.85 |
| 2030 | 75-79 years | 148.10 |
| 2031 | 75-79 years | 140.72 |
| 2032 | 75-79 years | 133.67 |
| 2033 | 75-79 years | 126.91 |
| 2034 | 75-79 years | 120.43 |
| 2035 | 75-79 years | 114.28 |
| 1990 | 80-84 years | 1557.08 |
| 1991 | 80-84 years | 1440.69 |
| 1992 | 80-84 years | 1330.91 |
| 1993 | 80-84 years | 1228.37 |
| 1994 | 80-84 years | 1134.09 |
| 1995 | 80-84 years | 1049.02 |
| 1996 | 80-84 years | 966.45 |
| 1997 | 80-84 years | 884.62 |
| 1998 | 80-84 years | 808.86 |
| 1999 | 80-84 years | 744.76 |
| 2000 | 80-84 years | 697.52 |
| 2001 | 80-84 years | 665.72 |
| 2002 | 80-84 years | 640.63 |
| 2003 | 80-84 years | 619.36 |
| 2004 | 80-84 years | 598.52 |
| 2005 | 80-84 years | 574.67 |
| 2006 | 80-84 years | 543.38 |
| 2007 | 80-84 years | 506.54 |
| 2008 | 80-84 years | 468.74 |
| 2009 | 80-84 years | 434.43 |
| 2010 | 80-84 years | 408.08 |
| 2011 | 80-84 years | 389.28 |
| 2012 | 80-84 years | 373.64 |
| 2013 | 80-84 years | 359.80 |
| 2014 | 80-84 years | 346.45 |
| 2015 | 80-84 years | 332.03 |
| 2016 | 80-84 years | 316.49 |
| 2017 | 80-84 years | 300.34 |
| 2018 | 80-84 years | 283.73 |
| 2019 | 80-84 years | 266.69 |
| 2020 | 80-84 years | 249.21 |
| 2021 | 80-84 years | 235.03 |
| 2022 | 80-84 years | 216.53 |
| 2023 | 80-84 years | 205.34 |
| 2024 | 80-84 years | 194.77 |
| 2025 | 80-84 years | 184.79 |
| 2026 | 80-84 years | 175.39 |
| 2027 | 80-84 years | 166.55 |
| 2028 | 80-84 years | 158.20 |
| 2029 | 80-84 years | 150.30 |
| 2030 | 80-84 years | 142.81 |
| 2031 | 80-84 years | 135.73 |
| 2032 | 80-84 years | 129.01 |
| 2033 | 80-84 years | 122.64 |
| 2034 | 80-84 years | 116.56 |
| 2035 | 80-84 years | 110.77 |
| 1990 | 85-89 years | 1609.49 |
| 1991 | 85-89 years | 1487.77 |
| 1992 | 85-89 years | 1372.94 |
| 1993 | 85-89 years | 1267.54 |
| 1994 | 85-89 years | 1170.68 |
| 1995 | 85-89 years | 1081.34 |
| 1996 | 85-89 years | 994.70 |
| 1997 | 85-89 years | 909.08 |
| 1998 | 85-89 years | 829.96 |
| 1999 | 85-89 years | 763.46 |
| 2000 | 85-89 years | 715.99 |
| 2001 | 85-89 years | 683.62 |
| 2002 | 85-89 years | 657.39 |
| 2003 | 85-89 years | 634.44 |
| 2004 | 85-89 years | 611.72 |
| 2005 | 85-89 years | 585.10 |
| 2006 | 85-89 years | 552.82 |
| 2007 | 85-89 years | 516.59 |
| 2008 | 85-89 years | 480.00 |
| 2009 | 85-89 years | 446.60 |
| 2010 | 85-89 years | 420.26 |
| 2011 | 85-89 years | 400.34 |
| 2012 | 85-89 years | 383.46 |
| 2013 | 85-89 years | 368.64 |
| 2014 | 85-89 years | 354.42 |
| 2015 | 85-89 years | 339.31 |
| 2016 | 85-89 years | 323.06 |
| 2017 | 85-89 years | 306.58 |
| 2018 | 85-89 years | 289.96 |
| 2019 | 85-89 years | 273.34 |
| 2020 | 85-89 years | 255.93 |
| 2021 | 85-89 years | 241.74 |
| 2022 | 85-89 years | 217.90 |
| 2023 | 85-89 years | 206.27 |
| 2024 | 85-89 years | 195.37 |
| 2025 | 85-89 years | 185.13 |
| 2026 | 85-89 years | 175.49 |
| 2027 | 85-89 years | 166.40 |
| 2028 | 85-89 years | 157.81 |
| 2029 | 85-89 years | 149.69 |
| 2030 | 85-89 years | 142.02 |
| 2031 | 85-89 years | 134.80 |
| 2032 | 85-89 years | 128.01 |
| 2033 | 85-89 years | 121.59 |
| 2034 | 85-89 years | 115.52 |
| 2035 | 85-89 years | 109.77 |
| 1990 | 90-94 years | 1654.79 |
| 1991 | 90-94 years | 1534.28 |
| 1992 | 90-94 years | 1421.52 |
| 1993 | 90-94 years | 1309.31 |
| 1994 | 90-94 years | 1202.43 |
| 1995 | 90-94 years | 1104.96 |
| 1996 | 90-94 years | 1013.51 |
| 1997 | 90-94 years | 926.40 |
| 1998 | 90-94 years | 848.10 |
| 1999 | 90-94 years | 782.60 |
| 2000 | 90-94 years | 734.52 |
| 2001 | 90-94 years | 701.06 |
| 2002 | 90-94 years | 673.90 |
| 2003 | 90-94 years | 650.83 |
| 2004 | 90-94 years | 629.71 |
| 2005 | 90-94 years | 605.78 |
| 2006 | 90-94 years | 574.63 |
| 2007 | 90-94 years | 538.42 |
| 2008 | 90-94 years | 500.92 |
| 2009 | 90-94 years | 466.22 |
| 2010 | 90-94 years | 438.42 |
| 2011 | 90-94 years | 418.21 |
| 2012 | 90-94 years | 401.44 |
| 2013 | 90-94 years | 386.77 |
| 2014 | 90-94 years | 372.71 |
| 2015 | 90-94 years | 357.86 |
| 2016 | 90-94 years | 341.36 |
| 2017 | 90-94 years | 324.14 |
| 2018 | 90-94 years | 306.69 |
| 2019 | 90-94 years | 288.98 |
| 2020 | 90-94 years | 270.07 |
| 2021 | 90-94 years | 255.08 |
| 2022 | 90-94 years | 222.95 |
| 2023 | 90-94 years | 210.49 |
| 2024 | 90-94 years | 198.85 |
| 2025 | 90-94 years | 187.95 |
| 2026 | 90-94 years | 177.74 |
| 2027 | 90-94 years | 168.17 |
| 2028 | 90-94 years | 159.20 |
| 2029 | 90-94 years | 150.79 |
| 2030 | 90-94 years | 142.89 |
| 2031 | 90-94 years | 135.45 |
| 2032 | 90-94 years | 128.44 |
| 2033 | 90-94 years | 121.81 |
| 2034 | 90-94 years | 115.54 |
| 2035 | 90-94 years | 109.63 |
| 1990 | 95+ years | 1707.48 |
| 1991 | 95+ years | 1595.95 |
| 1992 | 95+ years | 1490.08 |
| 1993 | 95+ years | 1386.96 |
| 1994 | 95+ years | 1288.93 |
| 1995 | 95+ years | 1198.28 |
| 1996 | 95+ years | 1114.42 |
| 1997 | 95+ years | 1031.94 |
| 1998 | 95+ years | 949.85 |
| 1999 | 95+ years | 878.00 |
| 2000 | 95+ years | 821.66 |
| 2001 | 95+ years | 780.03 |
| 2002 | 95+ years | 745.56 |
| 2003 | 95+ years | 716.18 |
| 2004 | 95+ years | 688.97 |
| 2005 | 95+ years | 659.38 |
| 2006 | 95+ years | 623.36 |
| 2007 | 95+ years | 582.67 |
| 2008 | 95+ years | 540.76 |
| 2009 | 95+ years | 502.13 |
| 2010 | 95+ years | 472.63 |
| 2011 | 95+ years | 450.96 |
| 2012 | 95+ years | 432.61 |
| 2013 | 95+ years | 415.88 |
| 2014 | 95+ years | 399.49 |
| 2015 | 95+ years | 382.61 |
| 2016 | 95+ years | 365.37 |
| 2017 | 95+ years | 348.07 |
| 2018 | 95+ years | 331.01 |
| 2019 | 95+ years | 313.31 |
| 2020 | 95+ years | 293.76 |
| 2021 | 95+ years | 277.48 |
| 2022 | 95+ years | 238.90 |
| 2023 | 95+ years | 225.17 |
| 2024 | 95+ years | 212.26 |
| 2025 | 95+ years | 200.13 |
| 2026 | 95+ years | 188.77 |
| 2027 | 95+ years | 178.12 |
| 2028 | 95+ years | 168.17 |
| 2029 | 95+ years | 158.87 |
| 2030 | 95+ years | 150.17 |
| 2031 | 95+ years | 142.01 |
| 2032 | 95+ years | 134.37 |
| 2033 | 95+ years | 127.20 |
| 2034 | 95+ years | 120.49 |
| 2035 | 95+ years | 114.17 |
